# A new genus and three new species of miniaturized microhylid frogs from Indochina (Amphibia: Anura: Microhylidae: Asterophryinae)

**DOI:** 10.24272/j.issn.2095-8137.2018.019

**Published:** 2018-04-28

**Authors:** Nikolay A. Poyarkov, Chatmongkon Suwannapoom, Parinya Pawangkhanant, Akrachai Aksornneam, Tang Van Duong, Dmitriy V. Korost, Jing Che

**Affiliations:** 1 Department of Vertebrate Zoology, Biological Faculty, Lomonosov Moscow State University, Moscow 119234, Russia; 2 Joint Russian-Vietnamese Tropical Research and Technological Center, Nghia Do, Cau Giay, Hanoi, Vietnam; 3 Division of Fishery, School of Agriculture and Natural Resources, University of Phayao, Phayao 56000, Thailand; 4 Department of Zoology, Faculty of Science, Kasetsart University, Bangkok 10900, Thailand; 5 Vietnam National Museum of Nature, Vietnam Academy of Science and Technology, Hanoi, Vietnam; 6 Petroleum Geology Department, Geological Faculty, Lomonosov Moscow State University, Moscow 119234, Russia; 7 State Key Laboratory of Genetic Resources and Evolution, Kunming Institute of Zoology, Chinese Academy of Sciences, Kunming Yunnan 650223, China; 8 Southeast Asia Biodiversity Research Institute, Chinese Academy of Sciences, Yezin Nay Pyi Taw 05282, Myanmar

**Keywords:** *Vietnamophryne***Gen. nov.**, *Vietnamophryne inexpectata***sp. nov.**, *Vietnamophryne orlovi***sp. nov.**, *Vietnamophryne occidentalis***sp. nov.**, *Siamophryne*, *Gastrophrynoides*, mtDNA, micro-CT scanning, Vietnam, Thailand, Herpetofauna, Amphibia, Biogeography, Taxonomy, Indochina

## Abstract

We report on the discovery of a new genus of microhylid subfamily Asterophryinae from northern and eastern Indochina, containing three new species. *Vietnamophryne* **Gen. nov.** are secretive miniaturized frogs (SVL<21 mm) with a mostly semi-fossorial lifestyle. To assess phylogenetic relationships, we studied 12S rRNA – 16S rRNA mtDNA fragments with a final alignment of 2 591 bp for 53 microhylid species. Morphological and osteological characters were analyzed using micro-CT scanning and used to describe the new genus. Results of phylogenetic analyses assigned the new genus into the mainly Australasian subfamily Asterophryinae as a sister taxon to the genus *Siamophryne* from southern Indochina. The three specimens collected from Gia Lai Province in central Vietnam, Cao Bang Province in northern Vietnam, and Chiang Rai Province in northern Thailand proved to be separate species, different both in morphology and genetics (genetic divergence 3.1%≤*P*≤5.1%). Our work provides further evidence for the “out of Indo-Eurasia” scenario for Asterophryinae, indicating that the initial cladogenesis and differentiation of this group of frogs occurred in the Indochina Peninsula. To date, each of the three new species of *Vietnamophryne* **Gen. nov.** is known only from a single specimen; thus, their distribution, life history, and conservation status require further study.

## INTRODUCTION

Frogs of the family Microhylidae form one of the most speciose groups of amphibians with pantropical distribution. Currently, some 642 species are recognized, inhabiting areas from the tropics and subtropics of Africa, Madagascar, Southern and Northern America, and South, East, and Southeast Asia to the islands of the Australasian archipelago and northernmost Australia (Frost, 2018). The basal split within Microhylidae is2 estimated to have occurred ∼65 Ma, coinciding with the Cretaceous-Paleogene boundary (Feng et al., 2017). The family Microhylidae is assumed to be of Gondwanan origin and is currently divided in 13 subfamilies, each of which are associated with a certain landmass derived from the breakup of Gondwana (De Sá et al., 2012; Kurabayashi et al., 2011; Peloso et al., 2016). Despite significant progress in understanding the evolutionary relationships within the family, the level of congruence between morphology-based and molecular phylogenetic hypotheses is still low and further changes in family- and genus-level taxonomy are required (De Sá et al., 2012; Kurabayashi et al., 2011; Matsui et al., 2011; Peloso et al., 2016; Pyron & Wiens, 2011; Rivera et al., 2017).

The subfamily Asterophryinae is the most speciose group within Microhylidae, currently consisting of 327 species inhabiting the tropical forests of northern Australia, New Guinea, and adjacent Australasian islands westwards to Sulawesi, southern Philippines, and crossing the Wallace line in Bali (Frost, 2018). The original biogeographic hypothesis for this subfamily suggested that the common ancestor of Asterophryinae dispersed to Australia via an Antarctic land bridge (Hill, 2009; Savage, 1973), where it diversified and subsequently dispersed to New Guinea and adjacent Australasian islands. However, based on multilocus phylogenetic analyses, Kurabayashi et al. (2011) demonstrated that the enigmatic genus *Gastrophrynoides* from Sundaland (Borneo and Malay Peninsula) belongs to the subfamily Asterophryinae as a sister-lineage with respect to all Australasian taxa, suggesting that the basal split of the subfamily may not have occurred in Gondwana, but instead on the Eurasian mainland. Thus, Kurabayashi et al. (2011) proposed an “out of Indo-Eurasia” biogeographic scenario for Asterophryinae, suggesting that its colonization route was from Asia to Australia, and not via Antarctica as suggested earlier.

In their work, Kurabayashi et al. (2011:9) predicted, that the “biogeographic findings on *Gastrophrynoides* imply the possible occurrence of further microhylid taxa with unexpected evolutionary backgrounds and give a basis for future paleontological and biogeographic studies of Asian anurans”. Our more recent work (Suwannapoom et al., 2018) reported on the unexpected discovery of *Siamophryne* — a striking troglophilous microhylid frog found in a limestone cave in Tenasserim (southern Thailand) — with phylogenetic analyses placing it as a sister lineage of *Gastrophrynoides*, further suggesting that mainland Southeast Asia likely served as a cradle of initial divergence and radiation of asterophryine frogs.

**Figure 1:**
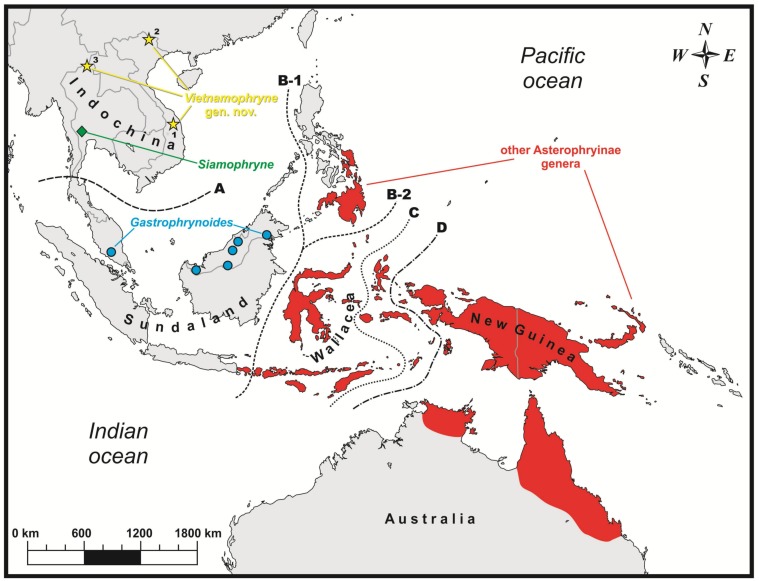
Known distribution of main Asterophryinae lineages and new genus *Vietnamophryne* Gen. nov. (yellow stars)

In 2016 and 2017, during field surveys in northern and eastern Indochina, we encountered three specimens of miniaturized frogs. Although these frogs were found in different localities in central and northern Vietnam and northern Thailand (Figure 1), all three specimens were superficially very similar to each other and found in similar microhabitats — soil or leaf litter under large tree logs or among plant roots. They were assigned to Microhylidae due to the presence of morphological characters diagnostic for the family: namely, lack of mandibular teeth, lack of parotoid glands, firmisternal pectoral girdle with non-overlapping epicoracoids, well-developed coracoids reaching midline of girdle and scapulae, large, cartilaginous sternum, and absence of clavicles and omosternum. Further morphological, osteological, and molecular analyses demonstrated that each of the three specimens represented a new species of a previously unknown lineage of frogs, assigned to the subfamily Asterophryinae and sister taxon to *Siamophryne*. We describe this new genus and three new species herein.

Biogeographic borders: A: Isthmus of Kra line, approximate biogeographic border between Sundaland and Indochina; B-1: Wallace line (after Huxley (1868)); B-2: Wallace line (after Mayr, 1944); C: Weber line; D: Lyddeker line. Most Asterophryinae genera inhabit Australasia, east of the Wallace line (red), and Bali; *Gastrophrynoides* is confined to Sundaland (Borneo and Malaysian Peninsula; blue circles); *Siamophryne* is known from a single locality in Tenasserim, southern Thailand (green diamond); *Vietnamophryne* **Gen. nov.** occurs in northern and eastern Indochina in Thailand and Vietnam. Localities: 1: *Vietnamophryne inexpectata* **sp. nov.**: Kon Chu Rang N.R., Gia Lai Province, Vietnam; 2: *Vietnamophryne orlovi* **sp. nov.**: Phia Oac-Phia Den N.P., Cao Bang Province, Vietnam; 3: *Vietnamophryne occidentalis* **sp. nov.**: Doi Tung Mt., Chiang Rai Province, Thailand.

## MATERIALS AND METHODS

### Sample collection

Field work was conducted from 23 May to 2 June 2016 in Kon Chu Rang Nature Reserve, Gia Lai Province, Tay Nguyen Plateau, central Vietnam (N14.506°, E108.542°; elevation 1 000 m a.s.l.); from 8 to 17 June 2017 in Phia Oac-Phia Den National Park, Cao Bang Province, northern Vietnam (N22.600°, E105.884°; elevation 1 200 m a.s.l.) and from 5 to 15 February and 4 to 8 April 2017 in the environs of Doi Tung Mt., Pong Ngam District, Chaing Rai Province, northern Thailand (N20.344°, E99.830°; elevations from 900 to 1 050 m a.s.l.). All fieldwork and collection permits are listed in the Acknowledgements. Geographic coordinates and elevation were obtained using a Garmin GPSMAP 60CSx (USA) and recorded in WGS84 datum. In total, three adult specimens (all males) were collected from three surveyed localities. The specimens were photographed in life and then euthanized using 20% benzocaine prior to fixation in 96% ethanol and subsequent storage in 70% ethanol. Tissue samples for genetic analysis were taken prior to preservation and stored in 95% ethanol. Specimens and tissues were subsequently deposited in the herpetological collections of the Zoological Museum of Moscow University (ZMMU, Moscow, Russia) and School of Agriculture and Natural Resources, University of Phayao (AUP, Phayao, Thailand).

### Laboratory methods

Total genomic DNA was extracted from ethanol-preserved femoral muscle tissue using standard phenol-chloroform-proteinase K (final concentration 1 mg/mL) extraction with subsequent isopropanol precipitation (as per Hillis et al., 1996 and Sambrook & Russell, 2001). The isolated DNA was visualized using agarose electrophoresis in the presence of ethidium bromide. The resulting DNA concentration in 1 μL was measured using a NanoDrop 2000 (Thermo Scientific, USA) and consequently adjusted to 100 ng DNA/μL.

We amplified mtDNA fragments, covering partial sequences of the 12S rRNA and 16S rRNA mtDNA genes and complete sequence of the tRNA${}^{\mathrm{Val}}$ mtDNA gene to obtain a 2 591-bp long continuous fragment of mtDNA. These mtDNA markers have been used for comprehensive phylogenetic studies on Microhylidae frogs (De Sá et al., 2012; Matsui et al., 2011; Peloso et al., 2016; Pyron & Wiens, 2011; Van Der Meijden et al., 2007; and references therein), including molecular taxonomic research on the subfamily Asterophryinae (Blackburn et al., 2013; Frost et al., 2006; Günther et al., 2010; Köhler & Günther, 2008; Kurabayashi et al., 2011; Oliver et al., 2013; Rittmeyer et al., 2012; Suwannapoom et al., 2018). PCR was performed in 20 μL reactions using 50 ng of genomic DNA, 10 nmol of each primer, 15 nmol of each dNTP, 50 nmol of additional MgCl_2_, Taq PCR buffer (10 mmol/L of Tris-HCl, pH 8.3, 50 mmol/L of KCl, 1.1 mmol/L of MgCl_2_ and 0.01% gelatin), and 1 U of Taq DNA polymerase. The PCR conditions as well as primers used for PCR procedures and sequencing followed Suwannapoom et al. (2018).

The PCR products were loaded onto 1.5% agarose gels in the presence of ethidium bromide. Visualization was carried out using agarose electrophoresis. If distinct bands were obtained, products were purified prior to cycle sequencing using 2 μL of ExoSapIt (Amersham, UK), diluted at a 1:4 ratio, per 5 μL of PCR product. The 10 μL sequencing reaction included 2 μL of template, 2.5 μL of sequencing buffer, 0.8 μL of 10 pmol primer, 0.4 μL of BigDye Terminator v3.1 Sequencing Standard (Applied Biosystems, USA), and 4.2 μL of water. The cycle sequencing reaction included 35 cycles with the following steps: 10 s at 96 °C, 10 s at 50 °C, and 4 min at 60 °C. Cycle sequencing products were then purified by ethanol precipitation. Sequencing was performed on an ABI 3730xl automated sequencer (Applied Biosystems, USA). The obtained sequences were deposited in GenBank under accession numbers MH004403–MH004406 (Table 1).

**Table 1:** Specimens and sequences of three new species of *Vietnamophryne* Gen. nov. from Indochina and outgroup representatives of Microhylidae and Rhacophoridae used in molecular analyses

Group	GenBank accession No.	Species	Specimen ID	Reference
Asterophryinae	DQ283195	*Aphantophryne pansa*	ABTC 49605	Frost et al., 2006
Asterophryinae	FR832625; FR832642	*Asterophrys (Asterophrys) turpicola*	ZMB 70537	Günther et al., 2010
Asterophryinae	KM509160	*Asterophrys (Metamagnusia) slateri*	PT-507	Peloso et al., 2016
Asterophryinae	FR832653; FR832636	*Asterophrys (Pseudocallulops) eurydactyla*	ZMB 70534	Günther et al., 2010
Asterophryinae	JN048979; JN049004	*Austrochaperina guttata*	LSUMZ 95008	Rittmeyer et al., 2012
Asterophryinae	KC822485	*Austrochaperina* sp.	BSFS 11377	Blackburn et al., 2013
Asterophryinae	EU100119; EU100235	*Barygenys exsul*	BPBM 20128	Köhler & Günther, 2008
Asterophryinae	KM509105	*Callulops robustus*	PT-506	Peloso et al., 2016
Asterophryinae	DQ283207	*Choerophryne* sp.	ABTC 47720	Frost et al., 2006
Asterophryinae	DQ283206	*Cophixalus sphagnicola*	ABTC 47881	Frost et al., 2006
Asterophryinae	DQ283208	*Copiula* sp.	AMS R124417	Frost et al., 2006
Asterophryinae	AB634647; AB634705	*Gastrophrynoides immaculatus*	UKMHC 279	Matsui et al., 2011
Asterophryinae	JX119248; JX119392	*Hylophorbus rufescens*	LSUMZ 94943	Oliver et al., 2013
Asterophryinae	JN048989; JN049014	*Mantophryne lateralis*	LSUMZ 92102	Rittmeyer et al., 2012
Asterophryinae	MH004403	*Vietnamophryne inexpectata* **Gen. et sp. nov.**	ZMMU A-5820	*This work*
Asterophryinae	MH004404	*Vietnamophryne orlovi* **Gen. et sp. nov.**	ZMMU A-5821	*This work*
Asterophryinae	MH004406	*Vietnamophryne occidentalis* **Gen. et sp. nov.**	ZMMU A-5822	*This work*
Asterophryinae	MG682553	Siamophryne*troglodytes*	ZMMU NAP-06651	Suwannapoom et al., 2018
Asterophryinae	FR832634; FR832635	*Oninia senglaubi*	ZMB 74608	Günther et al., 2010
Asterophryinae	KC822488	*Oreophryne anulata*	PNMCMNHH 1366	Blackburn et al., 2013
Asterophryinae	DQ283194	*Oreophryne brachypus*	ABTC 50081	Frost et al., 2006
Asterophryinae	AB634651; AB634709	*Oreophryne monticola*	MZBAmp 16265	Matsui et al., 2011
Asterophryinae	KC822489	*Oreophryne variabilis*	TNHC 58922	Blackburn et al., 2013
Asterophryinae	JN048996; JN049021	*Paedophryne amauensis*	BPBM 31882	Rittmeyer et al., 2012
Asterophryinae	JX119386; JX119242	*Sphenophryne (Sphenophryne) cornuta*	LSUMZ 94793	Oliver et al., 2013
Asterophryinae	DQ283209	*Sphenophryne (Genyophryne) thomsoni*	ABTC 49624	Frost et al., 2006
Asterophryinae	DQ283199	*Sphenophryne (Liophryne) rhododactyla*	ABTC 49566	Frost et al., 2006
Asterophryinae	EU100323; EU100207	*Sphenophryne (Oxydactyla) crassa*	BPBM 17061	Köhler & Günther, 2008
Asterophryinae	FR832655; FR832638	*Xenorhina* cf. *oxycephala*	ZMB 74628	Günther et al., 2010
Asterophryinae	KM509212	*Xenorhina obesa*	PT-529	Peloso et al., 2016
Chaperininae	AB598318; AB598342	*Chaperina fusca*	BORN 8478	Matsui et al., 2011
Dyscophinae	AB634648; AB634706	*Dyscophus guineti*	KUHE 33150	Matsui et al., 2011
Dyscophinae	AB634649; AB634707	*Dyscophus insularis*	KUHE 35001	Matsui et al., 2011
Gastrophryninae	AB634650; AB634708	*Gastrophryne olivacea*	KUHE 33224	Matsui et al., 2011
Kalophryninae	AB634642; AB634700	*Kalophrynus pleurostigma*	MZBAmp 15295	Matsui et al., 2011
Kalophryninae	AB634645; AB634703	*Kalophrynus subterrestris*	KUHE 53145	Matsui et al., 2011
Melanobatrachinae	KM509159	*Melanobatrachus indicus*	IND-18	Peloso et al., 2016
Microhylinae	AB201182; AB201193	*Glyphoglossus molossus*	KUHE 35182	Matsui et al., 2011
Microhylinae	AB634626; AB634684	*Glyphoglossus yunnanensis*	KUHE 44148	Matsui et al., 2011
Microhylinae	KP682314	*Kaloula rugifera*	–	Deng et al., 2016
Microhylinae	AB634634; AB634692	*Metaphrynella pollicaris*	KUZ-21655	Matsui et al., 2011
Microhylinae	AB634600; AB634658	*Microhyla annectens*	–	Matsui et al., 2011
Microhylinae	DQ512876	*Microhyla fissipes*	–	unpublished
Microhylinae	NC006406	*Microhyla heymonsi*	–	Zhang et al., 2005
Microhylinae	AB303950	*Microhyla okinavensis*	–	Igawa et al., 2008
Microhylinae	AB634616; AB634674	*Microhyla petrigena*	–	Matsui et al., 2011
Microhylinae	NC024547	*Microhyla pulchra*	–	Wu et al., 2016
Microhylinae	AB598317; AB598341	*Micryletta inornata*	KUHE 20497	Matsui et al., 2011
Microhylinae	AB634638; AB634696	*Micryletta steinegeri*	KUHE 35937	Matsui et al., 2011
Microhylinae	AB634636; AB634694	*Phrynella pulchra*	UKMHC 820	Matsui et al., 2011
Microhylinae	AB634633; AB634691	*Uperodon taprobanicus*	KUHE 37252	Matsui et al., 2011
Phrynomerinae	AB634652; AB634710	*Phrynomantis bifasciatus*	KUHE 33277	Matsui et al., 2011
Scaphiophryninae	AB634653; AB634711	*Scaphiophryne gottlebei*	KUHE 34977	Matsui et al., 2011
Rhacophoridae	AB202078	*Rhacophorus schlegelii*	-	Sano et al., 2005

–: Not available.

### Phylogenetic analyses

For phylogenetic analysis we used the 12S rRNA and 16S rRNA Microhylidae dataset of Suwannapoom et al. (2018) with the addition of the newly obtained sequences of Microhylidae **Gen. spp.** from Vietnam and Thailand. Data on sequences and specimens used in molecular analyses are summarized in Table 1. In total, sequences of the 12S rRNA and 16S rRNA mtDNA fragments of 53 microhylid representatives were included in the final analysis: including three samples of Microhylidae **Gen. spp.** from central and northern Vietnam and northern Thailand; 27 samples of the subfamily Asterophryinae (25 specimens of Australasian asterophryine genera and two specimens of *Gastrophrynoides* and *Siamophryne* from Sundaland and Tenasserim, respectively); 18 samples of Asian microhylids representing all major lineages of the family inhabiting this region (including subfamilies Microhylinae, Kalophryninae, Melanobatrachinae, and Chaperininae); and five outgroup sequences of non-Asian Microhylidae, including subfamilies Dyscophinae (Madagascar), Gastrophryninae (North America), Phrynomerinae (Africa), and Scaphiophryninae (Madagascar). An mtDNA sequence of *Rhacophorus schlegelii* (Günther) (Rhacophoridae) was used as a non-microhylid outgroup.

Nucleotide sequences were initially aligned using ClustalX 1.81 software (Thompson et al., 1997) with default parameters, and then optimized manually in BioEdit 7.0.5.2 (Hall, 1999) and MEGA 7.0 (Kumar et al., 2016). Mean uncorrected genetic distances (*P*-distances) between sequences were determined using MEGA 6.0. MODELTEST v3.06 (Posada & Crandall, 1998) was applied to estimate the optimal evolutionary models to be used for dataset analysis. The best-fitting model was the GTR+I+G model of DNA evolution, as suggested by the Akaike Information Criterion (AIC).

Phylogenetic trees were inferred using maximum likelihood (ML) and Bayesian inference (BI). The ML analysis was conducted using Treefinder (Jobb et al., 2004). Confidence in tree topology was tested by non-parametric bootstrap (BS) analysis with 1 000 replicates (Felsenstein, 1985). The BI analysis was conducted using MrBayes 3.1.2 (Huelsenbeck & Ronquist, 2001; Ronquist & Huelsenbeck, 2003). Metropolis coupled Markov chain Monte Carlo (MCMCMC) analyses were run with one cold chain and three heated chains for four million generations and were sampled every 1 000 generations. Five independent MCMCMC runs were performed and 1 000 trees were discarded as burn-in. Confidence in tree topology was assessed using posterior probability (PP) (Huelsenbeck & Ronquist, 2001). We regarded tree nodes with BS values of 75% or greater and PP values over 0.95 as sufficiently resolved, those with BS values between 75% and 50% (PP between 0.95 and 0.90) as tendencies, and those with BS values below 50% (PP below 0.90) as unresolved (Huelsenbeck & Hillis, 1993).

### Adult morphology

Sex of adult individuals was determined using gonadal dissection. All measurements were taken to the nearest 0.02 mm and subsequently rounded to a 0.1 mm precision from preserved specimens using a digital caliper under a light dissecting microscope. Measurements included the following 40 morphometric characters, as per Poyarkov et al. (2014) and Suwannapoom et al. (2018): (1) snout-vent length (SVL; length from tip of snout to cloaca); (2) head length (HL; length from tip of snout to hind border of jaw angle); (3) snout length (SL; length from anterior corner of eye to tip of snout); (4) eye length (EL; distance between anterior and posterior corners of eye); (5) nostril-eye length (N-EL; distance between anterior corner of eye and nostril center); (6) head width (HW; maximum width of head at level of mouth angles in ventral view); (7) internarial distance (IND; distance between central points of nostrils); (8) interorbital distance (IOD; shortest distance between medial edges of eyeballs in dorsal view); (9) upper eyelid width (UEW; maximum distance between medial edge of eyeball and lateral edge of upper eyelid); (10) forelimb length (FLL; length of straightened forelimb from limb base to tip of third finger); (11) lower arm and hand length (LAL; distance between elbow and tip of third finger); (12) hand length (HAL; distance between proximal end of outer palmar (metacarpal) tubercle and tip of third finger); (13) inner palmar tubercle length (IPTL; maximum distance between proximal and distal ends of inner palmar tubercle); (14) outer palmar tubercle length (OPTL; maximum diameter of outer palmar tubercle); (15) hindlimb length (HLL; length of straightened hindlimb from groin to tip of fourth toe); (16) tibia length (TL; distance between knee and tibiotarsal articulation); (17) foot and tibiotarsus length (FTL; length from tibiotarsal joint to end of fourth toe); (18) foot length (FL; distance between distal end of tibia and tip of fourth toe); (19) inner metatarsal tubercle length (IMTL; maximum length of inner metatarsal tubercle); (20) outer metatarsal tubercle length (OMTL; maximum length of outer metatarsal tubercle); (21) tympanum length, maximum tympanum diameter (TYD); (22) tympanum-eye distance (TED); (23–26) finger lengths (1–3FLO, 4FLI; for outer side (O) of first, inner side (I) of fourth, distance between tip and junction of neighboring finger); (27) first finger width (1FW), measured at distal phalanx; (28–30) finger disk diameters (2–4FDW); (31) first toe length (1TOEL), distance between distal end of inner metatarsal tubercle and tip of first toe; (32–35) second to fifth toe lengths (outer lengths for toes II–IV, inner length for toe V); (36–40) toe disk diameters (1–5TDW).

The morphological characters for comparison and data on states in other Microhylidae representatives were taken from: Burton (1986), Chan et al. (2009), Günther & Richards (2016), Günther (2009, 2017), Günther et al. (2010, 2012a, 2012b, 2014, 2016), Köhler & Günther (2008), Kraus & Allison (2003), Kraus (2010, 2011, 2013a, 2013b, 2014, 2016, 2017), Menzies & Tyler (1977), Parker (1934), Richards & Iskandar (2000), Richards et al. (1992, 1994), Rittmeyer et al. (2012), Suwannapoom et al. (2018), Zweifel (1972, 2000), and Zweifel (2003).

### Osteology

Micro-CT scanning protocols followed Suwannapoom et al. (2018). Micro-CT scanning was conducted at the Petroleum Geology Department, Faculty of Geology, Lomonosov Moscow State University using a SkyScan 1 172 desktop scanner (Bruker micro-CT, Kontich, Belgium) equipped with a Hamamatsu 10 Mp digital camera. Scanning was performed only for ZMMU A-5820. The specimen was mounted on a polystyrene baseplate and placed inside a hermetically sealed polyethylene vessel. Scans were conducted with a resolution of 3.7 μm at 100 keV voltages and a current of 100 mA with a rotation step of 0.2° in oversize mode in which four blocks of sub-scan data were connected vertically to obtain a general tomogram. Data processing was performed using Skyscan software: NRecon (reconstruction) and CTan/CTVol (3D model producing and imaging). Osteological terminology followed Scherz et al. (2017), Suwannapoom et al. (2018), and Trueb (1968, 1973). Micro-CT does not render cartilage, and therefore cartilage structures were omitted from the osteological descriptions.

## RESULTS

### Sequence variation

Final alignment of the studied 12S rRNA and 16S rRNA mtDNA fragments consisted of 2 591 sites: 1 059 sites were conserved and 1 408 sites were variable, of which 1 082 were parsimony-informative. The transition-transversion bias (R) was to 2.14. Nucleotide frequencies were A=34.21%, T=22.89%, C=24.95%, and G=17.95% (data given only for Microhylidae ingroup).

### Phylogenetic relationships

**Figure 2:**
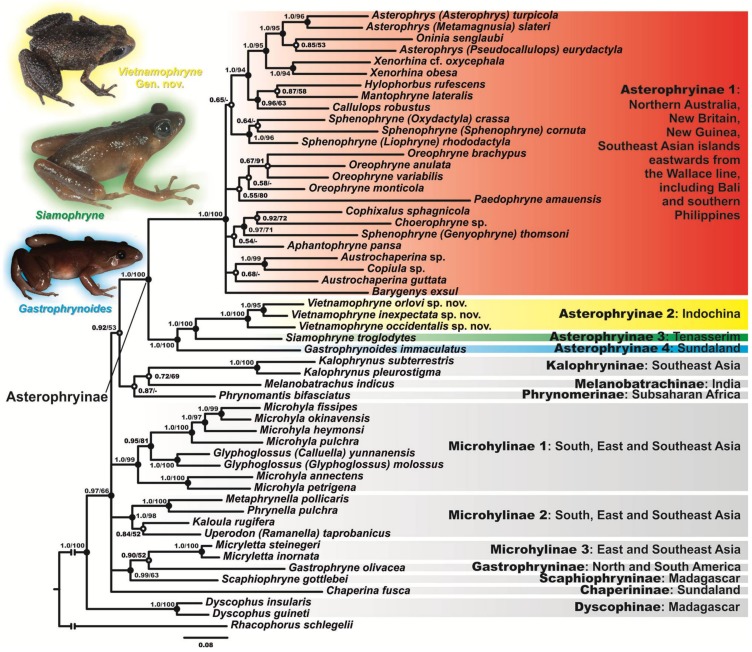
Bayesian inference dendrogram of Asterophryinae derived from analysis of 2 591-bp long 12S rRNA – 16S rRNA mtDNA gene fragments

Results of the phylogenetic analyses are shown in Figure 2. The BI and MI analyses resulted in essentially similar topologies. Though phylogenetic relationships between the subfamilies of Microhylidae remained essentially unresolved, high resolution was achieved among most major lineages of the subfamily Asterophryinae, with major nodes being sufficiently resolved (1.0/100; hereafter node support values are given for BI PP/ML BS, respectively; Figure 2). However, phylogenetic relationships within the Austro-Papuan radiation of Asterophryinae were poorly resolved with low or insignificant levels of support for major nodes. General topology of the phylogenetic relationships of the Microhylidae frogs was consistent with results reported in a number of recent studies (De Sá et al., 2012; Kurabayashi et al., 2011; Matsui et al., 2011; Peloso et al., 2016; Pyron & Wiens, 2011; Rivera et al., 2017; Suwannapoom et al., 2018; Van Der Meijden et al., 2007).

Voucher specimen IDs and GenBank accession numbers are given in Table 1. Sequence of *Rhacophorus schlegelii* was used as an outgroup. Numbers near branches represent posterior probability (PP) or bootstrap support values (BS, 1 000 replicates) for BI/ML inferences, respectively. Photos by N. A. Poyarkov and Y. Lee.

The BI tree (Figure 2) suggested the following genealogical relationships among the representatives of Microhylidae: monophyly of the subfamilies Dyscophinae, Kalophryninae, and Asterophryinae well-supported (1.0/100), monophyly of the subfamily Microhylinae not supported, and phylogenetic relationships among Microhylidae subfamilies unresolved.

The subfamily Asterophryinae consisted of the two major well-supported (1.0/100) reciprocally monophyletic clades:

(1) The Asterophryinae 1 or “core” Asterophryinae (Figure 2, in red) clade included all presently known Australasian genera of the subfamily inhabiting islands east of the Wallace line, tropical areas of northern Australia, and Bali (see line B1 in Figure 1; range of Asterophryinae 1 marked in red).

(2) The second clade included three Asterophryinae lineages inhabiting areas derived from the Eurasian landmass (mainland Southeast Asia and Sundaland) and included the genus *Gastrophrynoides* (Malay Peninsula and Borneo; lineage Asterophryinae 4 in Figure 2; range in Figure 1 marked in blue), recently discovered genus *Siamophryne* (Tenasserim in southern Thailand; lineage Asterophryinae 3; locality in Figure 1 marked in green), and the three newly discovered microhylids from central and northern Vietnam and northern Thailand (lineage Asterophryinae 2 on Figure 2; localities in Figure 1 marked in yellow).

Phylogenetic relationships among genera within the Asterophryinae 1 clade were essentially unresolved (Figure 2). *Cophixalus* was suggested as a sister lineage to *Choerophryne* with moderate support (0.92/72). Monophyly of the clade that included *Sphenophryne*, *Liophryne*, and *Oxydactyla* genera was strongly supported (1.0/96), thus supporting synonymy of the two latter genera with *Sphenophryne*, as suggested by Rivera et al. (2017). However, *Sphenophryne thomsoni* (Boulenger), previously assigned to the genus *Genyophryne*, was placed with significant node support (0.97/71) as a sister lineage to the clade that included *Cophixalus* and *Choerophryne* and was distantly related to the clade that included the remaining *Sphenophryne s. lato* taxa. Our data provided only weak support for monophyly of the genus *Oreophryne* (0.55/80). *Callulops* was identified as a sister lineage to *Mantophryne* and *Hylophorbus* (0.96/63). The monophyly of the clade that included *Asterophrys*, *Oninia*, and the formerly recognized genera *Metamagnusia* and *Pseudocallulops*, was strongly supported (1.0/95). The monophyly of the genus *Xenorhina* also showed high support (1.0/94).

Phylogenetic relationships among Asterophryinae clades 2–4 were well-resolved (Figure 2). Monophyly of the lineage Asterophryinae 2, joining three small microhylids from northern and eastern Indochina, was strongly supported (1.0/100); among them, the two Vietnamese samples from Cao Bang and Gia Lai provinces formed a strongly supported monophyletic group (1.0/95). The genus *Siamophryne* from Tenasserim (southern Thailand) was reconstructed as a sister lineage with respect to Asterophryinae 2 (1.0/100). The genus *Gastrophrynoides* from Sundaland was suggested as a sister-clade with respect to Indochinese lineages *Siamophryne* + Asterophryinae 2 with strong node support (1.0/100).

Our phylogenetic analyses indicated that the three newly discovered Microhylidae **Gen. sp.** from northern and eastern Indochina formed a monophyletic group, belonging to the mainly Australasian subfamily Asterophryinae *s. lato*, within which they were placed as a sister lineage to the genus *Siamophryne* (the only other asterophryine genus known from Indochina) with high levels of node support.

### Genetic distances

16S rRNA is a widely known molecular marker applied for biodiversity studies in amphibians (Vences et al., 2005a, 2005b; Vieites et al., 2009). The uncorrected genetic *P*-distances among and within the 12S rRNA – 16S rRNA gene fragments of the studied Asterophryinae genera are shown in Table 2. The genetic differentiation between the newly discovered Microhylidae **Gen. sp.** from northern and eastern Indochina and other Asterophryinae genera varied from 12.6% (between Microhylidae **Gen. sp.** from Cao Bang Province (Vietnam) and genus *Metamagnusia*) to 21.4% of substitutions (between Microhylidae **Gen. sp.** from Gia Lai Province (Vietnam) and genus *Callulops*). Genetic distances between Microhylidae **Gen. sp.** and its sister lineage *Siamophryne* varied from 12.6% to 15.1% of substitutions. These genetic divergences were high and corresponded well to genus level differentiation within Asterophryinae (Table 2). Genetic divergence between the three specimens of Microhylidae **Gen. sp.** was moderate and varied from 3.1% (between samples from Gia Lai Province of Vietnam and Chiang Rai Province of Thailand) to 5.1% (between Gia Lai and Cao Bang samples) of substitutions, slightly higher than the conventional threshold of species-level divergence in other groups of Anura (3.0% of divergence in the 16S rRNA gene according to Vences et al., 2005a, 2005b; Vieites et al., 2009).

### Taxonomy

Based on our phylogenetic analyses, the newly discovered miniaturized microhylid frogs from northern and eastern Indochina formed a monophyletic group, clearly distinct from all other members of Microhylidae for which comparable genetic data were available. This group was placed in the radiation of the subfamily Asterophryinae with strong support. Though the 12S rRNA – 16S rRNA mtDNA fragment sequences did not achieve full phylogenetic resolution for all lineages of the subfamily Asterophryinae, the phylogenetic relationships within our focal group, Asterophryinae lineages 2–4, were well-resolved. Our data strongly suggest that the three main lineages of Asterophryinae inhabiting Indochina and Sundaland were monophyletic, whereas the miniaturized Microhylidae **Gen. sp.** from northern Indochina were suggested as the sister-lineage of the genus *Siamophryne* from southern Indochina.

Subsequent analyses of osteology and external morphology (see below) strongly suggest that the recently discovered miniaturized Microhylidae **Gen. sp.** from northern and eastern Indochina represent a new previously undescribed genus with three new species, which we describe herein:

**Amphibia Linnaeus, 1758**
**Anura Fischer von Waldheim, 1813**
**Microhylidae Günther, 1858**
**Asterophryinae Günther, 1858**
***Vietnamophryne* Gen. nov.**

**Diagnosis:** Small-sized (14.2 mm<SVL<20.5 mm) member of the mainly Australasian subfamily Asterophryinae (family Microhylidae), with the following combination of morphological attributes: (1) both maxillae and dentaries eleutherognathine, no maxillary teeth; (2) vertebral column procoelous with eight presacral vertebrae lacking neural crests; (3) no cranial sagittal crest; (4) frontoparietals connected by long non-calcified suture; (5) nasals wide, calcified, not contacting medially; (6) vomeropalatines and neopalatine not expanded, not calcified (possibly, cartilaginous), vomerine spikes absent; (7) cultriform process of parasphenoid broad and short, abruptly obtuse anteriorly; (8) clavicles absent; (9) omosternum absent; (10) sternum small, non-calcified, completely cartilaginous, xiphisternum flat, rounded; (11) distinct dorsal crest present on urostyle at three-quarters of its length, absent on ilium; (12) terminal phalanges small, bobbin-shaped; (13) no disks on digits, digit tips rounded; (14) first finger reduced to nub or shortened, all phalanges present and ossified; (15) subarticular tubercles absent; (16) toe webbing absent; (17) tympanum distinct; (18) single transverse smooth palatal fold; (19) pupil round; (20) snout rounded, subequal to or shorter than eye length; (21) skin on dorsum warty to shagreened; and (22) semi-fossorial (mostly subterranean) lifestyle.

**Type species:** *Vietnamophryne inexpectata* **sp. nov.**

**Other included species:** *Vietnamophryne orlovi* **sp. nov.**; *Vietnamophryne occidentalis* **sp. nov.**

**Distribution:** To date, *Vietnamophryne* **Gen. nov.** is known only from three localities in northern and eastern Indochina: two localities in Vietnam (Gia Lai Province, Tay Nguyen Plateau of the central Annamite (Truong Son) Mountains and mountainous area in Cao Bang Province, northern Vietnam) and one locality in northern Thailand (limestone mountainous area in northern Chiang Rai Province) (Figure 1). This distribution pattern, joining the north-eastern part of Vietnam (Dong Bac), central Annamites (Tay Nguyen), and northern Thailand, suggests that members of the new genus may be found in other areas of northern and eastern Indochina, and its occurrence in adjacent regions of Laos and central-northern Vietnam is strongly anticipated.

**Table 2:** Uncorrected *P*-distances (percentages) between 12S rRNA – 16S rRNA sequences of *Vietnamophryne* Gen. nov. and other Asterophryinae genera included in phylogenetic analyses (below diagonal line) and standard error estimates (above diagonal line)

	Taxon	1	2	3	4	5	6	7	8	9	10	11	12	13	14	15	16	17	18	19	20	21	22	23	24	25
**1**	*Vietnamophryne inexpectata* **Gen. et sp. nov.**	—	1.1	1.1	1.9	2.3	2.5	2.0	1.7	2.0	2.2	2.2	2.4	2.1	2.2	2.1	2.1	2.1	2.1	2.3	1.7	2.2	1.9	1.9	2.3	1.9
**2**	*Vietnamophryne orlovi* **Gen. et sp. nov.**	5.1	—	1.0	1.9	2.3	2.6	2.1	1.9	2.2	2.2	2.6	2.7	2.3	2.4	2.3	2.1	2.1	2.1	2.5	1.8	2.3	2.1	2.1	2.3	2.1
**3**	*Vietnamophryne occidentalis* **Gen. et sp. nov.**	3.1	4.7	—	1.9	2.2	2.2	1.8	1.7	1.9	2.2	2.2	2.5	1.9	2.1	2.1	2.0	2.0	2.0	2.2	1.5	2.2	2.0	1.8	2.2	1.9
**4**	Siamophryne	15.1	12.6	14.8	—	2.1	2.3	2.2	1.7	2.2	2.2	2.3	2.3	2.1	2.1	2.2	2.1	2.3	2.1	2.1	1.9	2.2	1.9	2.3	2.3	2.1
**5**	*Gastrophrynoides*	17.5	16.5	16.8	17.2	—	2.4	2.4	1.9	2.3	2.6	2.5	2.8	2.4	2.4	2.4	2.2	2.5	2.4	2.4	2.0	2.1	2.0	2.0	2.4	2.3
**6**	*Aphantophryne*	19.6	20.9	19.9	17.5	17.9	—	2.0	1.6	2.5	2.2	2.2	2.4	2.1	2.2	2.1	2.0	2.4	2.0	2.2	1.8	2.1	2.0	2.1	2.1	2.0
**7**	*Asterophrys* s.str.	16.8	15.4	15.5	17.9	16.5	15.8	—	1.4	1.9	1.8	2.0	2.2	1.9	2.2	2.0	1.7	2.1	1.3	1.8	1.4	1.9	1.9	1.7	2.0	1.8
**8**	*Austrochaperina*	17.7	17.7	17.9	17.9	17.0	15.8	12.9	12.7	1.7	1.7	1.7	1.8	1.6	1.9	1.6	1.5	1.6	1.5	1.7	1.2	1.5	1.4	1.3	1.8	1.5
**9**	*Barygenys*	17.6	17.4	17.2	19.7	18.3	19.7	12.4	14.5	—	2.1	2.1	2.3	2.2	2.0	2.2	1.8	2.0	2.1	2.1	1.8	2.1	2.1	2.0	1.9	2.2
**10**	*Callulops*	21.4	19.8	21.4	20.0	17.6	16.6	12.8	15.5	16.3	—	2.0	2.3	2.0	2.3	1.6	2.0	1.7	2.0	2.2	1.7	1.9	2.1	1.8	1.8	2.0
**11**	*Choerophryne*	20.0	20.9	20.7	24.8	22.4	19.3	19.3	17.9	20.1	19.7	—	2.2	2.3	2.1	2.2	2.2	2.2	2.2	2.3	1.7	2.2	2.2	2.0	2.2	2.2
**12**	*Cophixalus*	19.0	19.0	20.0	20.7	21.0	17.9	16.2	16.9	16.3	17.0	20.4	—	2.1	2.3	2.5	2.1	2.7	2.4	2.5	1.8	2.2	2.3	2.3	2.2	2.3
**13**	*Copiula*	16.7	16.4	16.4	17.4	18.1	15.3	14.6	14.1	17.5	17.5	18.9	17.5	—	2.1	2.0	2.0	2.1	1.7	2.0	1.6	2.2	2.0	1.7	2.2	1.9
**14**	*Genyophryne*	16.6	15.0	16.2	17.9	17.9	16.2	15.9	16.6	15.2	19.7	18.0	17.3	17.5	—	2.4	1.9	2.4	2.2	2.2	1.8	2.3	2.1	2.0	2.2	2.1
**15**	*Hylophorbus*	17.2	15.4	17.2	18.2	18.6	17.2	14.4	14.9	19.0	13.4	17.2	19.0	14.6	18.6	—	2.1	1.6	2.0	2.2	1.6	2.0	2.1	1.9	2.2	1.8
**16**	*Liophryne*	17.2	16.9	16.5	15.8	15.8	14.8	12.0	14.4	13.8	12.4	18.6	15.2	13.2	16.2	13.7	—	2.1	1.7	2.0	1.4	1.8	1.9	2.0	1.6	1.8
**17**	*Mantophryne*	16.2	13.8	16.2	19.0	17.9	17.2	15.2	13.3	16.6	13.1	18.3	17.6	15.4	16.3	9.0	13.8	—	2.1	2.3	1.8	2.2	2.2	2.0	2.0	2.2
**18**	*Metamagnusia*	15.1	13.8	14.1	17.5	15.8	15.5	5.8	12.4	14.8	14.5	18.3	15.2	12.5	15.2	14.4	12.4	12.8	—	1.9	1.4	2.0	1.8	1.6	1.8	1.8
**19**	*Oninia*	19.0	17.8	17.6	17.6	18.3	17.6	17.2	17.9	18.3	20.4	21.1	21.8	17.9	19.7	17.2	15.5	18.0	15.9	—	1.7	1.9	2.1	2.1	2.1	1.9
**20**	*Oreophryne*	17.9	18.7	17.6	19.8	18.4	16.9	13.8	15.3	16.8	17.6	19.6	17.7	15.7	18.4	17.3	14.1	17.7	13.5	18.8	15.1	1.5	1.5	1.4	1.6	1.7
**21**	*Oxydactyla*	17.5	17.7	17.5	19.6	14.8	18.9	12.4	13.1	15.5	13.4	19.0	13.8	17.1	18.3	14.8	10.7	14.1	12.4	17.6	15.6	—	2.0	1.8	1.9	1.9
**22**	*Paedophryne*	18.6	18.6	18.6	19.3	16.9	17.6	14.8	15.2	17.6	18.3	21.8	18.3	18.2	17.3	19.0	15.5	17.3	12.8	20.4	16.7	14.8	—	2.0	2.1	2.0
**23**	*Pseudocallulops*	17.2	15.7	17.2	19.2	17.5	15.8	12.0	13.4	17.2	14.1	15.9	17.2	14.2	14.8	13.7	15.1	13.1	10.7	18.3	15.7	14.8	15.9	—	1.9	1.8
**24**	*Sphenophryne* s.str.	18.6	17.3	17.5	18.2	17.9	16.2	12.4	14.4	15.2	13.8	18.3	16.2	14.6	15.9	15.1	9.6	12.8	11.3	16.9	15.3	13.4	15.9	16.5	—	2.0
**25**	*Xenorhina*	17.5	17.3	17.7	18.6	18.2	15.6	14.1	14.2	16.6	14.5	19.7	17.5	16.3	18.7	16.7	15.1	16.5	13.2	19.6	17.0	16.6	17.9	13.8	16.2	14.0

Mean uncorrected intrageneric *P*-distances for the ingroup are shown in the diagonal in bold.

**Comparisons with other Asterophryinae genera:** Information on character states for other Asterophryinae genera is based on Parker (1934), Zweifel (1972, 2000), Menzies & Tyler (1977), Burton (1986), Zweifel (2003), Günther et al. (2010), Kraus (2010, 2017), Suwannapoom et al. (2018), and references therein. *Vietnamophryne* **Gen. nov.** can be distinguished from *Asterophrys* (including recently synonymized *Pseudocallulops* and *Metamagnusia*; Rivera et al., 2017), *Callulops*, *Mantophryne*, *Oninia*, and *Xenorhina* (including recently synonymized *Xenobatrachus* Peters & Doria) by eleutherognathine maxillae and dentaries (vs. symphignathine maxillae and dentaries in all these Asterophryinae genera), and from *Barygenys* (vs. symphignathine dentaries and eleutherognathine maxillae). *Vietnamophryne* **Gen. nov.** can be differentiated from genera *Aphantophryne* and *Cophixalus* by lack of distinct neural crests on presacral vertebrae (vs. well-developed neural crests on presacral vertebrae). *Vietnamophryne* **Gen. nov.** can be further distinguished from *Aphantophryne* by its eight presacral vertebrae (vs. seven). *Vietnamophryne* **Gen. nov.** can be distinguished from members of the genus *Sphenophryne s. lato* (including *Liophryne* and *Oxydactyla*) and *Austrochaperina* by absence of clavicles (vs. well-developed long and slender clavicles). *Vietnamophryne* **Gen. nov.** can be further distinguished from *Sphenophryne s. lato* by its lack of vomeropalatines (vs. broad vomeropalatines contacting each other medially, with post-choanal portion overlying palatine region). *Vietnamophryne* **Gen. nov.** can be diagnosed from *Sphenophryne s. stricto* (*S. cornuta* Peters & Doria) by smooth upper eyelid and semi-fossorial lifestyle (vs. spine-like projection on upper eyelid and arboreal lifestyle in *S. cornuta*). *Vietnamophryne* **Gen. nov.** can be further distinguished from the genus *Liophryne* (considered as a synonym of *Sphenophryne* by Rivera et al., 2017) by absence of finger disks (vs. small finger disks present). *Vietnamophryne* **Gen. nov.** can be further diagnosed from the genus *Oxydactyla* (coined as a synonym of *Sphenophryne* by Rivera et al., 2017) by F1 small or greatly reduced to nub (1FL≪122FL) (vs. F1 well-developed, 1FL≥122FL). *Vietnamophryne* **Gen. nov.** can be distinguished from the genus *Genyophryne* (coined as a synonym of *Sphenophryne* by Rivera et al., 2017) by absence of clavicles (vs. small clavicles present), lack of vomeropalatines and vomerine spikes (vs. expanded vomeropalatines with vomerine spikes), and F1 very small or reduced to nub, 1FL<122FL (vs. F1 well-developed, 1FL≥122FL). *Vietnamophryne* **Gen. nov.** can be further distinguished from *Austrochaperina* by lack of vomeropalatines (vs. vomeropalatines expanded). *Vietnamophryne* **Gen. nov.** differs from the genus *Paedophryne* by having all digit phalanges ossified (vs. cartilaginous phalanges in first digit), and eight presacral vertebrae (vs. seven). *Vietnamophryne* **Gen. nov.** can be diagnosed from the genus *Choerophryne* by lack of vomeropalatines (vs. palatine portions of vomeropalatines fused with broad sphenethmoids). *Vietnamophryne* **Gen. nov.** can be distinguished from the genus *Copiula* by lack of disks on fingers, but tiny disks on toes (vs. well-developed disks on fingers and toes) and absence of conspicuous rostral dermal gland (vs. rostral gland present). Semi-fossorial *Vietnamophryne* **Gen. nov.** can be easily distinguished from the mostly arboreal or terrestrial genus *Oreophryne* by its lack of toe webbing (vs. distinct toe webbing) and absence of vomeropalatines (vs. vomeropalatines expanded). *Vietnamophryne* **Gen. nov.** can be distinguished from *Hylophorbus* by comparatively better developed nasals (vs. poorly developed nasals), comparatively broad cultriform process of parasphenoid (vs. narrow cultriform process of parasphenoid), and F1 very small or reduced to nub, 1FL≪122FL (vs. F1 well-developed, 1FL≥122FL).

Among the Asterophryinae lineages inhabiting areas derived from the Eurasian landmass, *Vietnamophryne* **Gen. nov.** can be easily distinguished from the genus *Gastrophrynoides* (Malay Peninsula and Borneo) by snout rounded, length equal to or slightly more than eye length (vs. snout pointed, 2.5 times longer than eye; Figure 2), distinct tympanum (vs. tympanum obscured by skin; Figure 2), F1 very small or reduced to nub, 1FL≪122FL (vs. F1 well-developed, 1FL≥122FL), generally smaller body size, SVL≤20.5 mm (vs. SVL>20.0 mm), distinct crest on urostyle (vs. no crest on urostyle), bobbin-shaped terminal phalanges (vs. T-shaped terminal phalanges), single smooth palatal fold (vs. two palatal folds), comparatively broad cultriform process of parasphenoid (vs. narrow cultriform process of parasphenoid), and shagreened to warty skin (vs. completely smooth skin).

*Vietnamophryne* **Gen. nov.** can be easily distinguished from its sister genus *Siamophryne* (Tenasserim, south-western Thailand) by absence of finger disks (vs. large and wide finger disks; Figure 2), stout body habitus and generally smaller body size, SVL≤20.5 mm (vs. slender body habitus, SVL>20.0 mm), F1 very small or reduced to nub, 1FL≪122FL (vs. F1 well-developed, 1FL≥122FL), distinct crest on urostyle (vs. weak crest on urostyle), lack of clavicles (vs. small clavicles present), sternum fully cartilaginous (vs. anterior portion of sternum containing calcified cartilage), bobbin-shaped terminal phalanges (vs. large T-shaped terminal phalanges), single smooth palatal fold (vs. two palatal folds), comparatively broad cultriform process of parasphenoid (vs. narrow cultriform process of parasphenoid narrow), lack of vomeropalatines (vs. reduced but present), and shagreened to warty skin (vs. completely smooth skin).

Finally, the 12S-16S rRNA mtDNA fragment sequences for the new genus were markedly distinct from all sequences for Asterophryinae members for which homologous sequences were available (Figure 2, Table 2). 

**Comparisons with other Microhylidae genera inhabiting mainland Southeast Asia:** From other genera of Microhylidae inhabiting mainland Southeast Asia, all members of the genus *Vietnamophryne* **Gen. nov.** can be distinguished by a combination of the following characters: small body size (SVL≤21.0 mm); stout body habitus; externally distinct tympanum (vs. hidden tympanum in *Glyphoglossus*, *Microhyla*, *Micryletta*, *Kaloula*, *Phrynella*, *Metaphrynella*, and *Gastrophrynoides*); absence of subarticular tubercles (vs. subarticular tubercles of fingers greatly enlarged in *Phrynella* and *Metaphrynella*), absence of toe webbing or fringing on digits (vs. webbing or digit fringes present in *Microhyla*, *Phrynella*, and *Metaphrynella*); absence of tibiotarsal projection (vs. bony tibiotarsal projection present in *Chaperina*); lack of bony ridge along posterior border of each choana (vs. present in *Kaloula*); short rounded or obtuse snout (vs. long pointed snout 2.6–3.0 times eye diameter in *Gastrophrynoides*); and absence of disks on digits (vs. long limbs with digits bearing large disks, with those on fingers up to 2.5 times wider than penultimate phalanges in *Siamophryne*).

**Etymology:** The generic nomen *Vietnamophryne* is derived from “Vietnam”, the name of the country where the representatives of this genus were first recorded and where two of the three known species of the genus occur; and Greek noun “*phryne*” (φρ$\overset{´}{\upsilon }$νη; feminine gender), meaning “*toad*” in English; this root is often used in generic names in Asterophryinae frogs. Gender of the new genus is feminine. 

**Suggested common names:** We suggest the name “Indochinese Dwarf Frogs” as a common name of the new genus in English, “Nhái Lùn” as a common name of the new genus in Vietnamese, and “Eung Tham Khaera” as a common name of the new genus in Thai. 

***Vietnamophryne inexpectata*** **sp. nov.**

**Figure 3:**
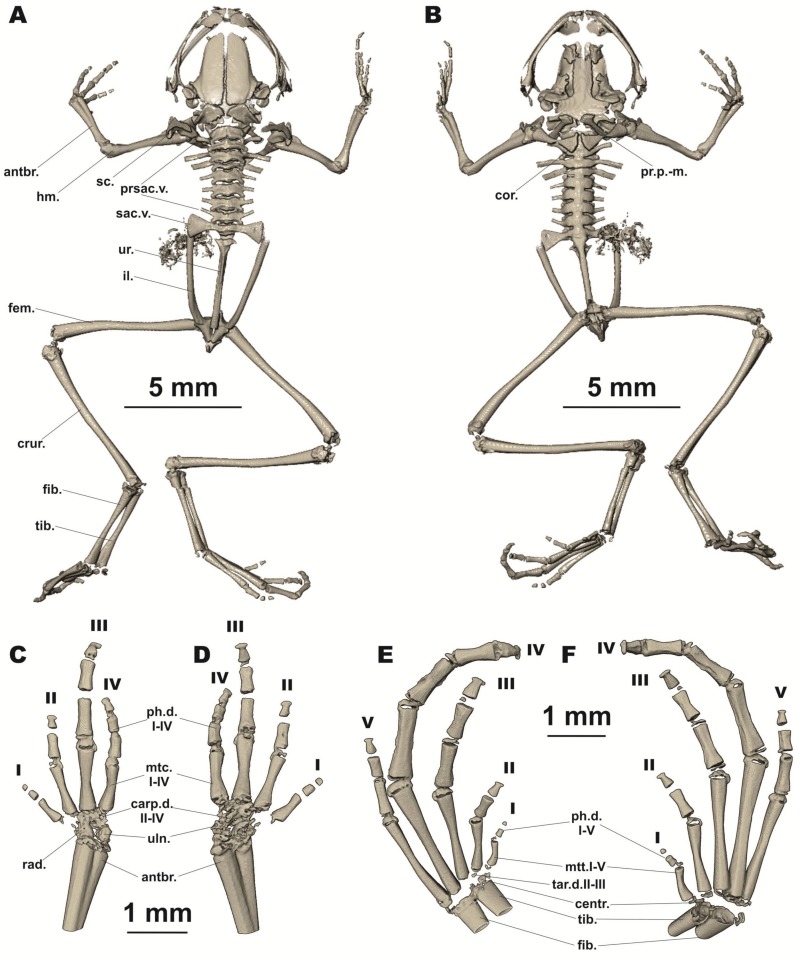
Osteology of *Vietnamophryne inexpectata* sp. nov. (male holotype, ZMMU A-5820), showing full skeleton in dorsal (A) and ventral views(B); right forelimb in dorsal (C) and palmar aspects (D); and right foot in thenar (E) and dorsal aspects (F)

**Figure 4:**
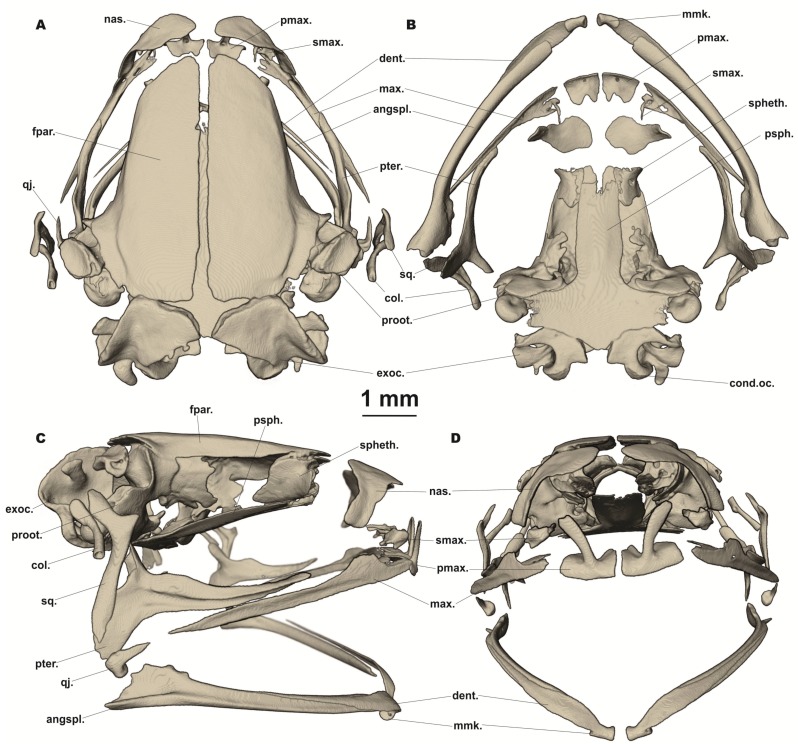
Osteology of *Vietnamophryne inexpectata* sp. nov. (male holotype, ZMMU A-5820), showing skull in dorsal (A); ventral (B); lateral (C); and frontal views (D)

Abbreviations: angspl.: angulosplenial; col.: columella; cond.oc.: occipital condylus; dent.: dentary bone; exoc.: exoccipital; fpar.: frontoparietal bone; max.: maxilla; mmk.: mentomeckelian bone; nas.: nasal bone; pmax.: premaxilla; proot.: prootic; psph.: parasphenoid; pter.: pterygoid; qj.: quadratojugal; smax.: septomaxilla; spheth.: sphenethmoid; sq.: squamosal. Scale bar: 1 mm.

**Figure 5:**
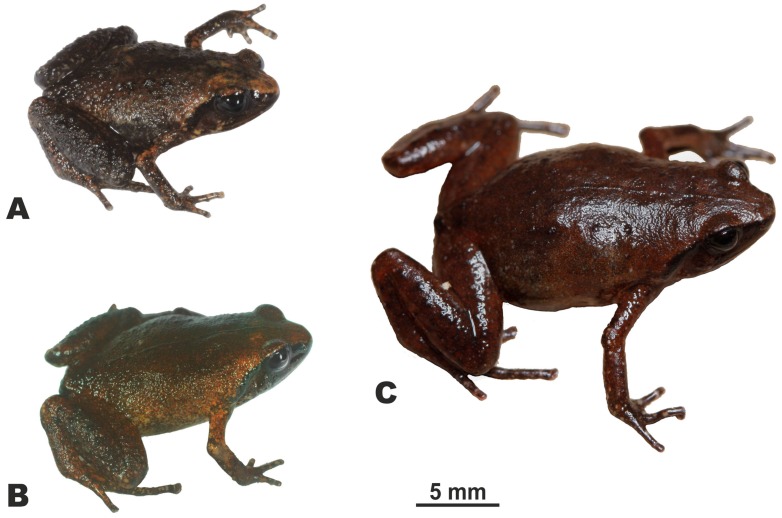
Three male holotypes of *Vietnamophryne* Gen. nov. species in life

**Figure 6:**
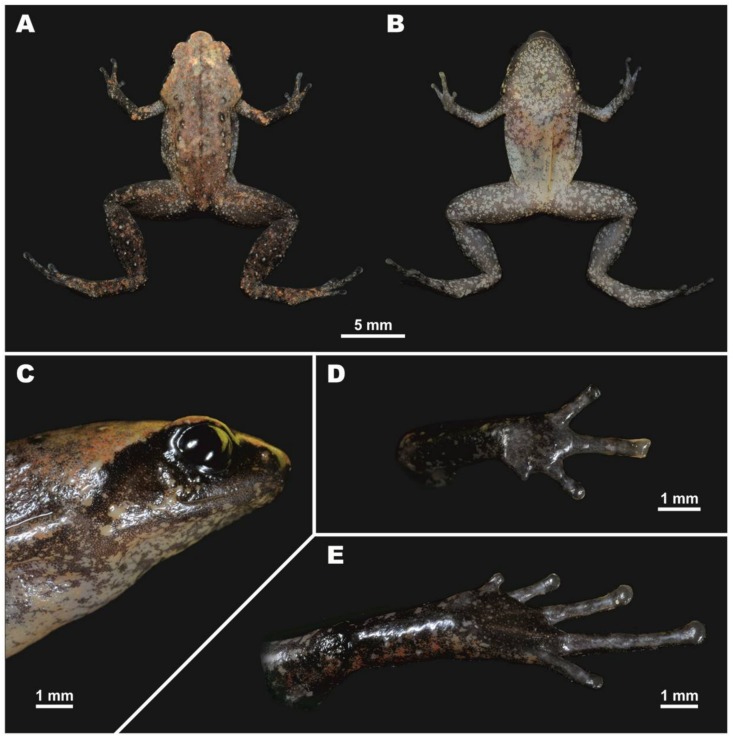
Male holotype of *Vietnamophryne inexpectata* sp. nov. (ZMMU A-5820) in life

Figure 3, Figure 4, Figure 5A, Figure 6; Table 3.

Digits numbered I–V. Abbreviations: antbr.: os antebrachii (radius+ulna); carp.d.II-IV: carpale distale F2-F4; centr.: centrale; cor.: coracoid bone; crur.: os cruris (tibia+fibula); fem.: femoral bone; fib.: fibulare; hm.: humeral bone; il.: ilium; mtc.I-IV: metacarpalia F1-F4; mtt.I-V: metatarsalia T1-T5; ph.d.I-IV: finger pahlanges F1-F4; ph.d.I-V: toe pahlanges T1-T5; pr.p.-m.: processus postero-medialis; prsac.v.: presacral vertebrae; rad.: radiale; sac.v.: sacral vertebra; sc.: scapula; tar.d.II-III: tarsale distale T2-T3; tib.: tibiale; uln.: ulnare; ur.: urostyle.

A: *Vietnamophryne inexpectata* **sp. nov.** (ZMMU A-5820); B: *Vietnamophryne orlovi* **sp. nov.** (ZMMU A-5821); C: *Vietnamophryne occidentalis* **sp. nov.** (ZMMU A-5822). Scale bar: 5 mm. Photos by N.A. Poyarkov (A, B) and P. Pawangkhanant (C).

A: Dorsal view; B: Ventral view; C: Lateral view of head; D: Palmar view of right hand; E: Thenar view of left foot. Photos by N. A. Poyarkov.

**Table 3:** Measurement data for holotypes of three new species of *Vietnamophryne* Gen. nov. from Indochina

Species	*V. inexpectata* sp. nov.	*V. orlovi* sp. nov.	*V. occidentalis* sp. nov.	Species	*V. inexpectata* sp. nov.	*V. orlovi* sp. nov.	*V. occidentalis* sp. nov.
Specimen ID	ZMMU A-5820	ZMMU A-5821	ZMMU A-5822	Specimen ID	ZMMU A-5820	ZMMU A-5821	ZMMU A-5822
	Holotype	Holotype	Holotype		Holotype	Holotype	Holotype
**Sex**	Male	Male	Male	**Sex**	Male	Male	Male
**1. SVL**	14.2	15.4	20.5	**21. TYD**	1.1	0.9	1.0
**2. HL**	5.4	6.7	6.9	**22. TED**	0.5	0.7	0.4
**3. SL**	1.8	2.5	2.1	**23. 1FL**	0.3	0.6	0.8
**4. EL**	1.9	1.8	2.5	**24. 2FL**	1.0	1.2	1.9
**5. N-EL**	0.9	2.0	1.4	**25. 3FL**	1.7	1.7	3.6
**6. HW**	5.4	5.8	6.8	**26. 4FL**	1.1	1.0	2.1
**7. IND**	1.5	1.8	2.4	**27. 1FW**	0.2	0.2	0.4
**8. IOD**	1.6	1.9	2.3	**28. 2FDD**	0.3	0.3	0.6
**9. UEW**	0.9	0.8	1.1	**29. 3FDD**	0.3	0.4	0.7
**10. FLL**	7.4	8.2	12.9	**30. 4FDD**	0.3	0.3	0.6
**11. LAL**	5.9	5.7	9.7	**31. 1TOEL**	0.4	0.7	1.0
**12. HAL**	3.2	3.2	5.6	**32. 2TOEL**	1.4	1.6	2.2
**13. IPTL**	0.5	0.6	0.7	**33. 3TOEL**	2.4	3.1	3.8
**14. OPTL**	0.5	0.7	0.6	**34. 4TOEL**	3.9	4.1	5.8
**15. HLL**	21.4	22.1	28.8	**35. 5TOEL**	1.6	1.8	2.8
**16. TL**	7.2	7.1	10.0	**36. 1TDD**	0.3	0.3	0.5
**17. FTL**	9.6	11.0	14.6	**37. 2TDD**	0.4	0.5	0.6
**18. FL**	6.6	7.1	8.2	**38. 3TDD**	0.5	0.6	0.8
**19. IMTL**	0.5	0.7	0.9	**39. 4TDD**	0.6	0.7	0.9
**20. OMTL**	–	–	–	**40. 5TDD**	0.4	0.4	0.6

For abbreviations see Materials and Methods. All measurements are in mm. –: Not available.

**Figure 7:**
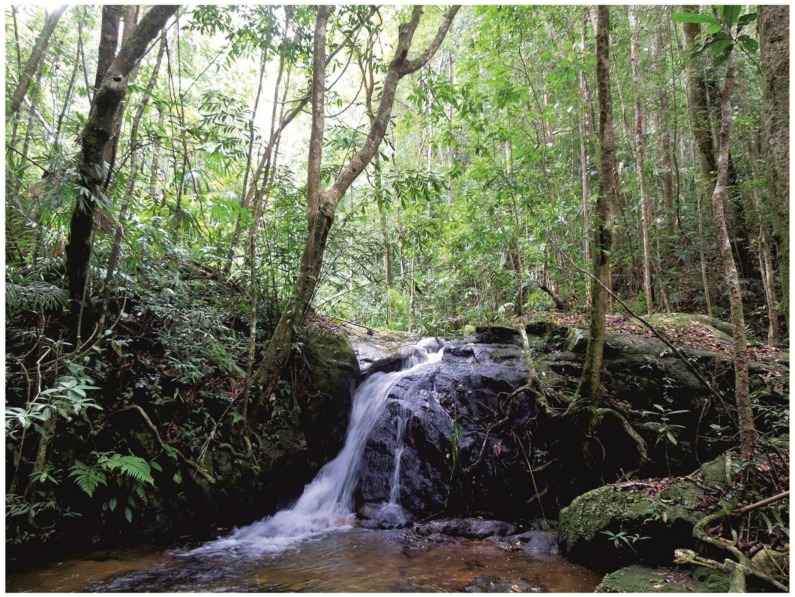
Habitat at type locality of *Vietnamophryne inexpectata* sp. nov. in Kon Chu Rang Nature Reserve, Gia Lai Province, Vietnam (Photo by A.V. Alexandrova)

**Holotype:** ZMMU A-5820, adult male in good state of preservation, from a primary montane tropical forest in Kon Chu Rang Nature Reserve, Gia Lai Province, Tay Nguyen Plateau, central Vietnam (N14.506°, E108.542°; elevation 1 000 m a.s.l.); collected on 31 May 2016 by Nikolay A. Poyarkov at 2100 h from soil under a large ca. 2-m long rotten log approximately 7 m from a small cascading stream (Figure 7). 

**Diagnosis:** Assigned to the genus *Vietnamophryne* **Gen. nov.** based on morphological characteristics and phylogenetic position (see Diagnosis of the new genus and Results). From other congeners *Vietnamophryne inexpectata* **sp. nov.** can be distinguished by the following combination of morphological characters: (1) miniaturized body size, SVL of single male 14.2 mm; (2) body habitus stout, FLL/SVL and HLL/SVL ratios 51.8% and 151.8%, respectively; (3) head as long as wide, HW/HL ratio 101.1%; (4) snout short, obtuse in dorsal view, rounded in lateral view, subequal to eye length (96.8% of eye length); (5) eye medium-sized, eye length/snout-vent length ratio 13%; eye to nostril distance 6.3% of SVL; (6) tympanum comparatively large and rounded, 7.9% of SVL; well separated from eye (TED/SVL ratio 3.6%); (7) tips of all digits rounded, not expanded in F1–F4, T1, T2, and T5, weakly expanded in T3 and T4; (8) first finger (F1) reduced to nub, less than one-third of F2 length (1FL/2FL ratio 29.2%); terminal phalanx of F1 reduced to tiny rounded ossification, relative finger lengths: I<II<IV<III, relative toe lengths: I<II<V<III<IV; (9) subarticular tubercles under fingers and toes weak, indistinct; (10) outer metatarsal tubercle absent, inner metatarsal tubercle small, rounded (3.5% of SVL); (11) skin of ventral surface completely smooth, skin of dorsal and lateral surfaces shagreened anteriorly, distinctly warty posteriorly with large flat tubercles or pustules finely scattered on posterior dorsum and dorsal surface of hindlimbs; (12) dorsomedial vertebral skin ridge indistinct, discernable only on dorsal surface of head; (13) dorsally grayish-brown with small reddish speckles anteriorly, darker tubercles posteriorly; lateral sides of head dark brown with beige mottling; ventrally gray-beige with weak gray marbling.

**Description of holotype:** Measurements of holotype are given in Table 3. Holotype in life is shown in Figure 5A and Figure 6. Body miniaturized, with SVL 14.2 (hereafter all measurements in mm), in good state of preservation; ventral surface of left thigh dissected 1.5 mm and partial femoral muscles removed. Body habitus stout (Figure 5A), head as long as wide (HL/HW 101.1%); snout short, obtuse in dorsal view (Figure 6A), rounded in profile (Figure 6C), subequal to eye diameter (SL/EL 96.8%); eyes medium-sized (EL/SVL 13.0%), slightly protuberant in dorsal and lateral views (Figure 6A, C), pupil round, horizontal (Figure 6C); dorsal surface of head slightly convex, canthus rostralis distinct, rounded; loreal region weakly concave; nostril rounded, lateral, located almost same distance from tip of snout and eye; tympanum well discerned, circular, comparatively large (TL/SVL ratio 7.9%), located distantly from eye (TED/SVL ratio 3.6%), tympanic rim not elevated above skin of temporal area, supratympanic fold present, glandular; vomerine teeth and spikes absent, single transverse palatal fold with smooth edge present across palate anteriorly to pharynx, tongue spatulate and free behind, lacking papillae, and vocal sac opening not discernable.

Forelimbs comparatively short, about one-third of hindlimb length (FLL/HLL 34.3%); hand shorter than lower arm, almost one-third of forelimb length (HAL/FLL 34.3%); fingers short, slender, round in cross-section, first finger reduced to nub, length comprising less than one-third of second finger (1FL/2FL 29.6%); relative finger lengths: I<II<IV<III (Figure 6D). Finger webbing and dermal fringes on fingers absent. First fingertip rudimentary, slightly protuberant as nub. Tips of three outer fingers II–IV rounded, not dilated, finger disks absent, terminal grooves absent; longitudinal furrow on dorsal surface of fingers absent; subarticular tubercles under fingers indistinct; nuptial pad absent; two palmar (metacarpal) tubercles: inner palmar tubercle small, rounded; outer palmar tubercle rounded with indistinct borders, slightly shorter than inner palmar tubercle (IPTL/OPTL 120.0%); palmar surface smooth, supernumerary palmar tubercles absent.

Hindlimbs comparatively short and thick, tibia length half of snout-vent length (TL/SVL 50.1%); tibiotarsal articulation of adpressed limb reaching eye level; foot slightly shorter than tibia (FL/TL 90.9%); relative toe lengths: I<II<V<III<IV; tarsus smooth, tarsal fold absent; tips of toes rounded, tips of toes III and IV slightly dilated (Figure 6E), terminal grooves on toes absent; toes rounded in cross-section, dermal fringes on toes absent; toe webbing absent between all toes; subarticular tubercles under toes indistinct; single metatarsal tubercle: inner metatarsal tubercle rounded, flattened (IMTL/SVL ratio 3.5%).

Skin on anterior dorsal and dorsolateral surfaces shagreened with numerous small flat tubercles (Figure 6A); tubercles larger and more prominent on posterior parts of dorsum, sacral area, and dorsal surfaces of hindlimbs; dorsal surface of forelimbs smooth with few small tubercles on forearm; upper eyelids and supratympanic folds with rows of enlarged tubercles forming flat glandular ridge; ventral sides of trunk, head, and limbs completely smooth (Figure 6B); weak indistinct dermal ridge present on midline of dorsal surface, running from tip of snout to scapular area (Figure 5A; Figure 6A).

**Coloration of holotype in life:** Dorsum grayish-brown, anteriorly light brown, posteriorly darker, with small reddish speckling anteriorly (Figure 6A); tubercles on sacral area, posterior parts of dorsum, and dorsal surfaces of hindlimbs dark gray with whitish pustules in middle; upper eyelids with tiny reddish speckles, two dorsolateral rows of darker tubercles running from scapular area toward vent; dorsal surfaces of forearms dark brown with red-brown blotches; dorsal surfaces of hindlimbs dark brown with rare reddish spots and dark gray to whitish tubercles and pustules; lateral sides of head dark brown with beige mottling present in tympanic area and mouth corners (Figure 6C); canthus rostralis ventrally dark brown, dorsally reddish-brown; supratympanic fold with whitish glandular tubercles; ventrally gray-beige with weak gray marbling, more scarce on belly, denser on chest, throat, and ventral surfaces of limbs (Figure 6B); fingers and toes dorsally dark brown with indistinct dark brown or reddish blotches, ventrally uniform gray (Figure 6D, E). Pupil round, black, iris uniform black (Figure 6C).

**Coloration of holotype in preservative:** Coloration pattern unchanged after preservation in ethanol for two years; however, dorsal coloration changed to grayish-brown and ventral surface of chest, belly, and limbs turned light gray.

**Osteological characteristics:** Osteological description is based on microtomographic data from male holotype. Main skeletal features are shown in Figure 3. Details of skull morphology are presented in Figure 4.

Skull clearly wider than long (Figure 3). Frontoparietals separate along entire length, longer than broad, narrower anteriorly than posteriorly, connected medially with long non-calcified suture, lacking sagittal crest, clearly separated from exoccipital by distinct suture posteriorly (Figure 4A). Exoccipitals separate, not contacting medially, sculptured laterally. Nasals large, not meeting at midline, lacking posterior ramus, with gently rounded ventrolateral processes, chondrified peripherally, separated from sphenethmoid (Figure 4A). Sphenethmoid poorly ossified only laterally, chondrified anteriorly, ventrally, and dorsally (Figure 4B). Prootics partially chondrified, with distinct dorsal crest (Figure 4C). Squamosal boomerang-shaped, well ossified, distally chondrified, articulating on lateral surface of prootic (Figure 4C). Columella large, centrally ossified (Figure 4C), distally chondrified, bent and barely pointing to otic area medially; tympanic annulus completely chondrified. Premaxilla with slender, well-ossified dorsal process not reaching nasal; labial process of premaxilla well ossified (Figure 4D). Maxilla largely chondrified, ossified in central and anterior parts. Upper jaw with eleutherognathine condition: anterior ends of maxillaries not reaching labial portions of well-developed premaxillaries (Figure 4D). Quadratojugal mostly cartilaginous, ossified only in posterior portion. Vomers possibly completely chondrified plates, lacking teeth or lateral processes; septomaxilla well ossified (Figure 4B). Mentomeckelians ossified, connected to dentaries and each other by strips of cartilage (Figure 4B). Lower jaw with eleutherognathine condition: dentaries not fused (Figure 4D). Parasphenoid smooth; cultriform process of parasphenoid rather broad, abruptly terminating at middle of sphenethmoid with distinct anterior notch (Figure 4B). Hyoid plate completely cartilaginous; posteromedial processes strongly ossified, elongated, notably enlarged and widened at proximal ends, chondrified at distal ends (Figure 3B).

Eight nonimbricate procoelous presacral vertebrae (PSV), stout, length approximately one-seventh to one-third of width; first presacral vertebra longer than posterior vertebrae, vertebrae width not changing posteriorly; all except first with wide diapophyses; transverse processes with chondrified tips, longer anteriorly (3d PSV with longest transverse processes), decreasing in length progressively to posterior (Figure 3A, B). Diapophyses of vertebrae PSV2, PSV7, and PSV8 oriented anteriad, those of PSV6 straight, and those of PSV3 to PSV5 oriented posteriad. Neural crests on PSV absent. Sacrum with notably expanded diapophyses (diapophyses length ca. 35% of sacrum width). Urostyle with well-pronounced dorsal crest running about 80% of shaft; ilia smooth, lacking dorsal crest (Figure 3A).

Coracoids, scapulae, and suprascapulae present; first two fully ossified; suprascapulae largely chondrified. Coracoids robust with narrow distal ends oriented anteriad; proximal ends greatly expanded, centrally notably narrowed (Figure 3B). Omosternum absent and clavicles absent. Sternum completely cartilaginous.

Hand bones (Figure 3C, D) with three poorly calcified carpal elements: carpale distale I chondrified, carpale distale II–IV fused into single large element, partially chondrified; prepollex chondrified; radiale large, partially calcified; ulnare rounded, partially calcified. Metacarpals short, distally and proximally chondrified, medially calcified; hand phalangeal formula: 2-2-3-3; all phalanges ossified; distal phalanx of finger I tiny, rudimentary, rounded (Figure 3C, D); terminal phalanges of fingers II–IV small, bobbin-shaped, notably narrower than penultimate phalanges (Figure 3C, D). Tarsal elements of foot mostly chondrified (Figure 3E, F), tiny ossifications present within generally cartilaginous tarsale distale II–III and central; prehallux chondrified. Metatarsals fully ossified medially, partially ossified distally, mostly chondrified proximally; metatarsals longer and relatively more massive than metacarpals; foot phalangeal formula: 2-2-3-4-3; all phalanges ossified medially, chondrified distally and proximally (Figure 3E, F). Terminal phalanges of all toes small, bobbin-shaped; notably narrower than penultimate phalanges on all toes (Figure 3E, F).

**Natural history notes:** Our knowledge on the biology of *Vietnamophryne inexpectata* **sp. nov.** is scarce. The single new species specimen was recorded in primary polydominant tropical montane evergreen forests of Tay Nguyen Plateau at an elevation of ca. 1 000 m a.s.l.. It was found during heavy rain at 2 100 h in wet soil at the bottom of a 20-cm deep hollow formed after a large 2-m long rotten tree log was turned over. The new species location was situated approximately 7 m from a small cascading stream (Figure 7). The frog was hiding among soil and leaf litter, suggesting that the new species has a semi-fossorial (subterranean) lifestyle or at least spends a considerable portion of its life hiding in leaf litter and under logs. The forest where the new species was recorded has a multi-layered canopy and heavy undergrowth, predominated by large trees of the families Podocarpaceae (*Dacrydium elatum*, *Dacrycarpus imbricatus*), Magnoliaceae, Burseraceae (*Canarium* sp.), Myrtaceae (*Syzygium* sp.), Hamamelidaceae (*Rhodoleia* sp., *Exbucklandia* sp.), Lauraceae (*Litsea* sp.), Fagaceae (*Lithocarpus* sp.), and Sterculiaceae (*Scaphium* sp.) (Figure 7).

Despite intensive fieldwork, no additional specimens of the new species were encountered either on the ground or in leaf litter over a 7-d period, suggesting a secretive biology for this frog. Diet and reproductive biology of the new species remain unknown. No calling activity was recorded during the survey. The male specimen was active at an air temperature of 21 °C with 100% humidity. The male possessed a pair of well-developed testes.

Other species of anurans recorded syntopically at the type locality included *Ingerophrynus galeatus* (Günther, 1864), *Kurixalus banaensis* (Bourret, 1939), *Rhacophorus annamensis* Smith, 1924, *Rhacophorus rhodopus* Liu & Hu, 1960, *Rh. robertingeri* Orlov, Poyarkov, Vassilieva, Ananjeva, Nguyen, Nguyen & Geissler, 2012, *Rana johnsi* Smith, 1921, *Microhyla pulverata* Bain & Nguyen, 2004, *Leptolalax* cf. *ardens* Rowley, Tran, Le, Dau, Peloso, Nguyen, Hoang, Nguyen & Ziegler, 2016, and *Ophryophryne hansi* Ohler, 2003.

**Comparisons:** For discrimination from other Microhylidae genera occurring in Indochina, see “Comparisons with other Microhylidae genera inhabiting mainland Southeast Asia” above.

*Vietnamophryne inexpectata* **sp. nov.** can be distinguished from its congeners based on the following morphological attributes. The new species can be distinguished from *Vietnamophryne orlovi* **sp. nov.** (inhabiting Cao Bang Province, northern Vietnam, described below) by warty skin on posterior and shagreened skin on anterior dorsum (vs. mostly smooth skin, slightly shagreened posteriorly, lacking enlarge tubercles), grayish-beige ventral coloration with gray marbling (vs. bright lemon-yellow belly with dark brown marbling), F1 reduced to nub, 1FL/2FL 29.6% (vs. F1 well-developed, 1FL/2FL 47.9%), head length almost equal to head width, HW/HL 101.1% (vs. head longer than wide, HW/HL 86.5%), snout length subequal to eye length, SL/EL 96.8% (vs. snout notably longer than eye length, SL/EL 141.3%), slightly larger tympanum, TYD/EL 60.5% (vs. TYD/EL 47.5%), and eye to nostril distance twice as short as eye length, N-EL/EL 48.1% (vs. N-EL/EL 109.5%).

*Vietnamophryne inexpectata* **sp. nov.** can be discriminated from *Vietnamophryne occidentalis* **sp. nov.** (inhabiting Chiang Rai Province, northern Thailand, described below) by the following combination of morphological characters: smaller body size, SVL 14.2 mm in single male holotype (vs. larger SVL 20.5 in single male holotype), warty skin on posterior and shagreened skin on anterior parts of dorsum (vs. mostly smooth skin with rare flat tubercles), grayish-beige ventral coloration with gray marbling (vs. bright orange-red belly with sparse dark brown marbling), F1 reduced to nub, 1FL/2FL 29.6% (vs. F1 well-developed, 1FL/2FL 42.7%), slightly larger tympanum, TYD/EL 60.5% (vs. TYD/EL 41.5%), and slightly shorter forelimb, FLL/SVL 51.7% (vs. comparatively longer forelimb, FLL/SVL 62.7%).

**Distribution and biogeography:** At present, *Vietnamophryne inexpectata* **sp. nov.** is known only from its type locality in montane tropical forest in Kon Chu Rang Nature Reserve, Gia Lai Province, central Vietnam at an elevation of ca. 1 000 m a.s.l.. The discovery of this secretive species in montane forests of other parts of Tay Nguyen Plateau at similar elevations in central Vietnam (Kon Tum, Quang Nam, Quang Ngai and Thua Thien-Hue provinces) and possibly in adjacent Laos is highly anticipated.

**Conservation status:** To date, the new species is known only from a single specimen, likely due to its secretive biology. The range and population status of *Vietnamophryne inexpectata* **sp. nov.** are unknown and further survey efforts in other parts of Tay Nguyen Plateau are required to understand its distribution and life history. Given the available information, we suggest *Vietnamophryne inexpectata* **sp. nov.** be considered as a Data Deficient (DD) species following IUCN’s Red List categories (IUCN Standards and Petitions Subcommittee, 2016).

**Etymology:** The specific name “*inexpectata*” is a Latin adjective in the nominative singular meaning “unexpected”; referring to the surprising discovery of this frog species in 2016, which belongs to the mainly Australasian subfamily Asterophryinae; until recently (Suwannapoom et al., 2018) members of Asterophryinae were not recorded from mainland Southeast Asia or eastern Indochina.

**Suggested common names.** We recommend the following common names for the new species: “Tay Nguyen Dwarf Frog” (English) and “Nhái Lùn Tây Nguyên” (Vietnamese).

***Vietnamophryne orlovi*** **sp. nov.**

**Figure 8:**
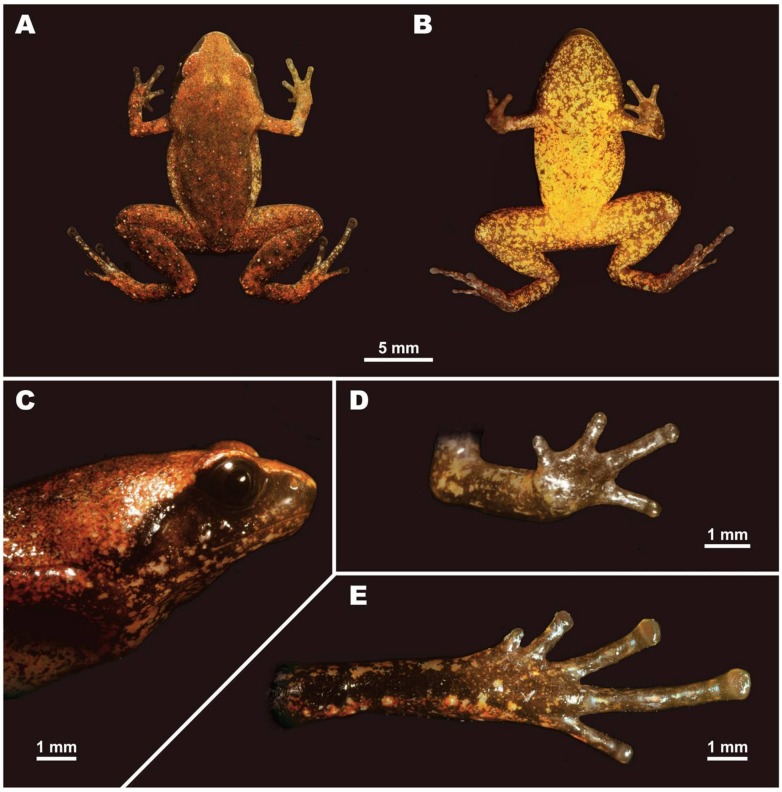
Male holotype of *Vietnamophryne orlovi* sp. nov. (ZMMU A-5821) in life

**Figure 9:**
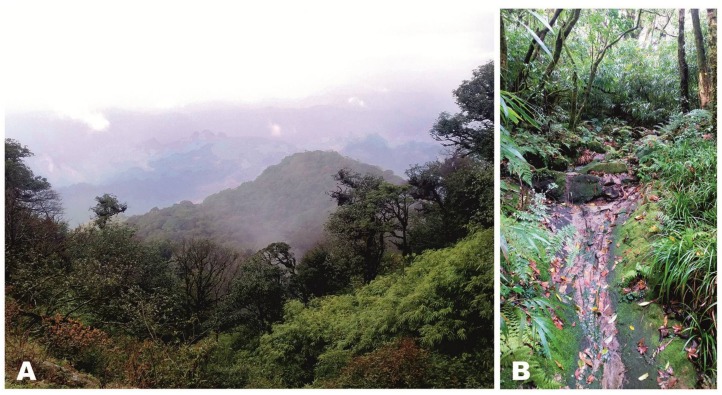
Macrohabitat (A) and microhabitat (B) at type locality of *Vietnamophryne orlovi* sp. nov. in Phia Oac-Phia Den N.P., Cao Bang Province, Vietnam (Photos by Le Xuan Son)

Figure 5B, Figure 8, Figure 9; Table 3.

A: Dorsal view; B: Ventral view; C: Lateral view of head; D: Palmar view of left hand; E: Thenar view of left foot. Photos by N. A. Poyarkov.

**Holotype:** ZMMU A-5821, adult male in good state of preservation, from a primary montane subtropical forest on the southern slopes of Phia Oac Mt., Phia Oac-Phia Den National Park, Cao Bang Province, northern Vietnam (N22.600°, E105.884°; elevation 1 200 m a.s.l.); collected on 9 June 2017 by Nikolay A. Poyarkov at 2300 h from soil in the roots of a tree fern on a steep mountain slope (Figure 9A), ca. 20 m from a small cascading stream (Figure 9B).

**Diagnosis:** Assigned to the genus *Vietnamophryne* **Gen. nov.** based on morphological attributes and phylogenetic position in mtDNA genealogy (see Diagnosis of the new genus and Results). From other congeners *Vietnamophryne orlovi* **sp. nov.** can be distinguished by the following combination of morphological traits: (1) miniaturized body size, SVL of single male 15.4 mm; (2) body habitus stout, FLL/SVL and HLL/SVL ratios 53.3% and 143.4%, respectively; (3) head longer than wide, HW/HL ratio 86.5%; (4) snout comparatively long, rounded in dorsal and lateral views, snout length greater than eye length (SL/EL ratio 141.3%); (5) eye medium-sized, eye length/snout-vent length ratio 11.6%; eye to nostril distance 12.7% of SVL; (6) tympanum comparatively small, rounded, 5.5% of SVL; well separated from eye (TED/SVL ratio 4.2%); (7) tips of all digits rounded, not expanded in F1–F4, T1, T2, and T5, weakly expanded in T3 and T4; (8) first finger (F1) well developed, half of F2 length (1FL/2FL ratio 47.9%), relative finger lengths: I<IV<II<III, relative toe lengths: I<II<V<III<IV; (9) subarticular tubercles under fingers and toes weak, indistinct; (10) outer metatarsal tubercle absent, inner metatarsal tubercle small, rounded (4.2% of SVL); (11) skin of ventral surface completely smooth, skin of dorsal and lateral surfaces smooth anteriorly, somewhat shagreened posteriorly with small flat pustules loosely scattered on posterior dorsum and dorsal surface of hindlimbs; (12) dorsomedial vertebral skin ridge distinct, discernable only on midline of dorsum and head; (13) dorsally reddish-brown, pustules on posterior dorsum whitish; lateral sides of head dark brown with whitish mottling; ventrally lemon-yellow with fine brown marbling.

**Description of holotype:** Measurements of holotype are given in Table 3. Holotype in life is shown in Figure 5B and Figure 8. Body miniaturized, SVL 15.4, in good state of preservation; ventral surface of left thigh dissected 1.6 mm and partial femoral muscles removed. Body habitus stout (Figure 5B), head notably longer than wide (HL/HW 86.5%); snout comparatively long, rounded in dorsal view (Figure 8A), truncate in lateral view (Figure 8C), snout length greater than eye length (SL/EL ratio 141.3%); eyes medium-sized (EL/SVL ratio 11.6%); eye to nostril distance 12.7% of SVL; eyes slightly protuberant in dorsal and lateral views (Figure 8A, C), pupil round, horizontal (Figure 8C); dorsal surface of head slightly convex, canthus rostralis distinct, rounded; loreal region concave; nostril rounded, lateral, located closer to tip of snout than to eye; tympanum well discernable, circular, comparatively small (TL/SVL ratio 5.5%), located distantly from eye (TED/SVL ratio 4.2%), tympanic rim not elevated above skin of temporal area, supratympanic fold present, distinct, glandular; vomerine teeth and spikes absent, single transverse palatal fold with smooth edge present across palate anteriorly to pharynx, tongue spatulate and free behind, papillae on tongue absent, vocal sac opening absent.

Forelimbs comparatively short, around one-third of hindlimb length (FLL/HLL 37.2%); hand shorter than lower arm, almost one-third of forelimb length (HAL/FLL 38.7%); fingers short, round in cross-section, first finger well developed, half of length of second finger (1FL/2FL 47.9%); relative finger lengths: I<IV<II<III (Figure 8D). Finger webbing and dermal fringes on fingers absent. First finger tip rounded, first finger well developed. Tips of three outer fingers II–IV rounded, not dilated, finger disks absent, terminal grooves absent; longitudinal furrow on dorsal surface of fingers absent; subarticular tubercles under fingers indistinct; nuptial pad absent; two palmar tubercles: inner palmar tubercle small, rounded; outer palmar tubercle rounded, slightly longer than inner palmar tubercle (IPTL/OPTL 90.9%); palmar surface smooth, supernumerary palmar tubercles absent.

Hindlimbs short and thick, tibia length less than half of snout-vent length (TL/SVL 46.0%); tibiotarsal articulation of adpressed limb reaching eye level; foot length equal to tibia length (FL/TL 100.7%); relative toe lengths: I<II<V<III<IV; tarsus smooth, tarsal fold absent; tips of toes rounded, tips of toes III and IV slightly dilated (Figure 8E), terminal grooves or dermal fringes on toes absent; toes rounded in cross-section; toe webbing absent between all toes; subarticular tubercles under toes indistinct; single metatarsal tubercle: inner metatarsal tubercle rounded, flattened (IMTL/SVL ratio 4.2%).

Skin on anterior dorsal and dorsolateral surfaces smooth, shagreened on posterior dorsum and dorsal surfaces of hindlimbs; small flat tubercles loosely scattered on sacral area and dorsal surfaces of limbs (Figure 8A); dorsal surface of forelimbs smooth; upper eyelids smooth, supratympanic folds with low glandular ridges; ventral sides of trunk, head and limbs completely smooth (Figure 8B); well-developed distinct dermal ridge present on midline of head dorsal surface, running from tip of snout to sacral area (Figure 5B; Figure 8A).

**Coloration of holotype in life:** Dorsum reddish-brown, anteriorly orange-brown, numerous small red speckles densely scattered on dorsal surfaces of head, body, and limbs (Figure 8A); posterior parts of dorsum and dorsal surfaces of hindlimbs with tiny whitish pustules; upper eyelids and canthus rostralis with narrow whitish stripe formed by tiny flat tubercles: stripe from snout tip toward eye along canthus rostralis, continuing to superciliary area and indistinct on supratympanic fold; dorsal surfaces of forearms brick-red; dorsal surfaces of hindlimbs reddish-brown with numerous reddish spots and rare whitish tubercles and pustules; lateral sides of head dark brown with whitish mottling on upper jaw and mouth corners (Figure 8C); canthus rostralis ventrally dark brown, dorsally with whitish stripe continuing to upper eyelid; supratympanic fold with reddish glandular tubercles lacking white stripe; ventrally bright lemon-yellow with weak dark brown marbling, marbling more scarce on ventral part of thighs and vent area, denser anteriorly toward chest and throat area (Figure 8B); fingers and toes dorsally gray-brown with indistinct reddish blotches, ventrally gray-brown with irregular beige or yellowish blotches (Figure 8D, E). Pupil round, black, iris uniform dark brown (Figure 8C).

**Coloration of holotype in preservative:** Coloration pattern unchanged after preservation in ethanol for one year; however, dorsal coloration changed to dark gray yellow tint on ventral surfaces of body and limbs faded to gray-beige.

**Natural history notes:** The biology of *Vietnamophryne orlovi* **sp. nov.** is unknown. The only encountered specimen of the new species was discovered at 2300 h under heavy rain in soil around the roots of cf. *Dicranopteris* sp. ferns (Gleicheniaceae, Gleicheniales), approximately 10 cm underground; the frog burrow was located on a steep slope of Phia Oac Mt. (Figure 9A), ca. 20 m from a small cascading stream (Figure 9B) at an elevation of ca. 1 200 m a.s.l. and air temperature of 17 °C. Thus, this species may exhibit a semi-fossorial lifestyle. Despite thorough search efforts, no additional individuals were recorded during a 10-d field survey in Phia Oac-Phia Den National Park, possibly due to the secretive biology of this frog. Diet and reproductive biology of *Vietnamophryne orlovi* **sp. nov.** remain unknown. No calling activity was recorded during the survey. The male possessed a pair of well-developed testes.

The polydominant subtropical forests in Phia Oac-Phia Den National Park at elevations of 1 200–1 400 m a.s.l. show thick bamboo undergrowth and are dominated by trees from the families Fagaceae (*Lithocarpus*, *Castanopsis*), Sapindaceae (*Acer*), Platanaceae (*Platanus*), Elaeocarpaceae (*Elaeocarpus*), Ericaceae (*Rhododendron*), Lauraceae (*Cinnamomum*), and Theaceae (*Schima*), with thick layers of moss and numerous epiphytic plants (Orchidaceae, Ericaceae, Pteridophyta) (Figure 9).

In Phia Oac, under the influence of the monsoon tropical climate of northeast Vietnam with cold winters and summer rains, the mean annual temperature, precipitation, and humidity are 20.6 °C, 1 718 mm, and 83.4%, respectively (Averyanov et al., 2003; Le, 2005). Unusually for northern Vietnam, the temperature can fall below freezing and snow is not rare in December and January. The dry season extends from November to April, with a mean precipitation of 295 mm (17.2% of total annual rainfall); the rainy season runs from May to November, with peak rainfall in July and August and mean rainfall of 1 423 (82.8% of total annual rainfall; Le, 2005). These conditions support a variety of forest types, particularly low to high montane broadleaf evergreen forests (Tran et al., 2014). Currently, vegetation covers approximately 84% of the total area of Phia Oac, though mostly consists of secondary forests or plantations. Mature (primary) and undisturbed forests are found only above 1 000 m a.s.l. (Tran et al., 2014).

Other species of amphibians recorded syntopically with the new species at the type locality include *Tylototriton ziegleri* Nishikawa, Matsui & Nguyen, 2013, *Raorchestes parvulus* (Boulenger, 1893), *Kurixalus odontotarsus* (Ye & Fei, 1993), *Gracixalus gracilipes* (Bourret, 1937), *Gracixalus jinxiuensis* (Hu, 1978), *Polypedates mutus* (Smith, 1940), and *Ophryophryne microstoma* Boulenger, 1903.

**Comparisons:** For comparisons with other members of the family Microhylidae occurring in Indochina, see “Comparisons with other Microhylidae genera inhabiting mainland Southeast Asia” above. For comparisons with *Vietnamophryne inexpectata* **sp. nov.** see the “Comparisons” section above.

*Vietnamophryne orlovi* **sp. nov.** can be distinguished from *Vietnamophryne occidentalis* **sp. nov.** (known from Chiang Rai Province, northern Thailand, described below) based on the following combination of morphological features: smaller body size, SVL 15.4 mm in single male holotype (vs. larger SVL 20.5 in single male holotype), lemon-yellow belly with dark brown marbling (vs. bright orange-red belly with sparse dark brown marbling), head longer than wide, HW/HL 86.5% (vs. head length almost equal to head width, HW/HL 99.0%), snout notably longer than eye length, SL/EL 141.3% (vs. snout length notably shorter than eye length, SL/EL 85.5%), eye to nostril distance almost equal to eye length, N-EL/EL 109.5% (vs. eye to nostril distance twice as short as eye length, N-EL/EL 55.2%), and slightly shorter forelimb, FLL/SVL 53.2% (vs. comparatively longer forelimb, FLL/SVL 62.7%).

**Distribution and biogeography:** At present, *Vietnamophryne orlovi* **sp. nov.** is known only from the type locality on Phia Oac Mt., in the montane subtropical forest of Phia Oac-Phia Den National Park, Cao Bang Province, northern Vietnam at an elevation of ca. 1 200 m a.s.l.. Phi Oac Mt. is the highest peak of the Ngan Son-Yen Lac Mountain Ridge located in northeastern Vietnam (Cao Bang, Bak Kan, and Thai Nguyen provinces); the occurrence of this species in other montane forest areas of the Ngan Son-Yen Lac Mountain Ridge at similar elevations is considered likely.

**Conservation status:** At present, *Vietnamophryne orlovi* **sp. nov.** is only known from a single specimen, possibly due to the secretive semi-fossorial biology of the species. The distribution and population status of *Vietnamophryne orlovi* **sp. nov.** are unknown and additional surveys in other areas of the Dong Bac (north-east) region of Vietnam are essential for elucidating the biology of the new species and clarifying its distribution. Given the available information, we suggest *Vietnamophryne orlovi* **sp. nov.** be considered as a Data Deficient (DD) species following IUCN’s Red List categories (IUCN Standards and Petitions Subcommittee, 2016).

**Etymology:** The specific name “*orlovi*” is a Latinized patronymic in genitive singular; the name of the new species is given in honor of Dr. Nikolai L. Orlov (ZISP, St. Petersburg, Russia) for recognition of his outstanding contribution to the knowledge of herpetofauna of Indochina.

**Suggested common names:** We recommend the following common names for the new species: “Orlov’s Dwarf Frog” (English) and “Nhái Lùn Đông Bac” (Vietnamese).

***Vietnamophryne occidentalis*** **sp. nov.**

**Figure 10:**
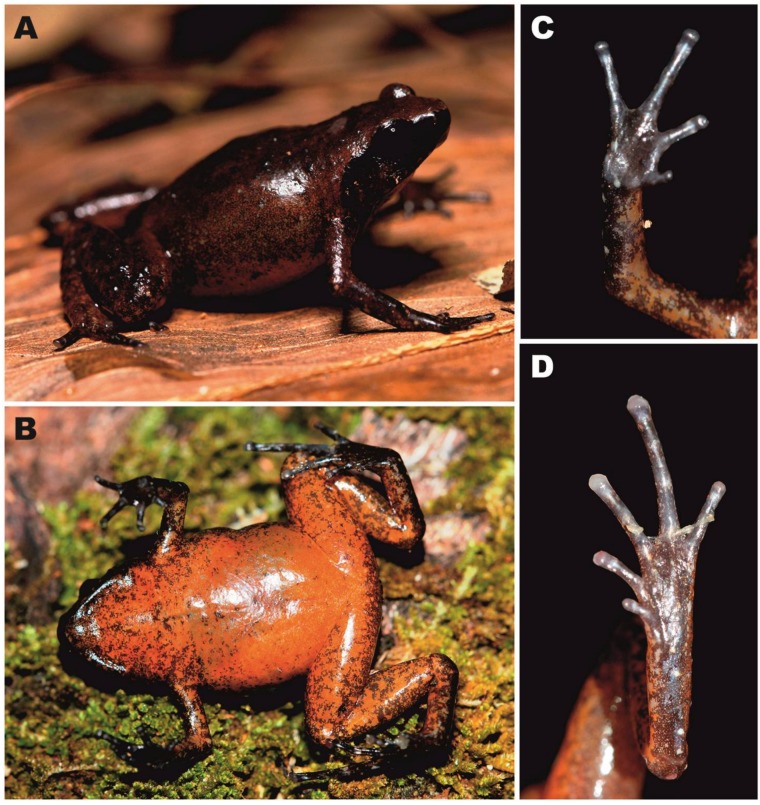
Male holotype of *Vietnamophryne occidentalis* sp. nov. (ZMMU A-5822) in life

**Figure 11:**
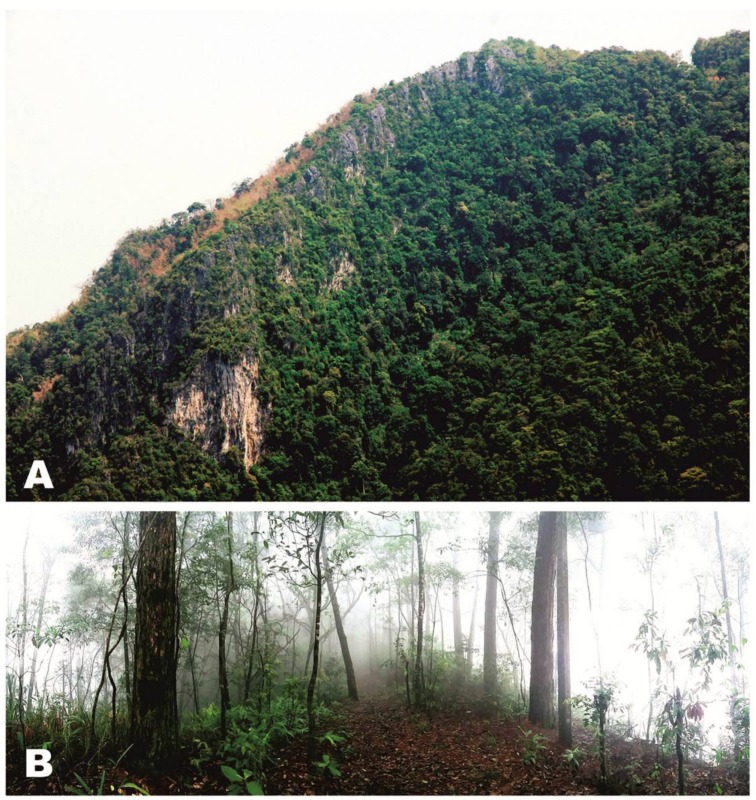
Macrohabitat (A) and microhabitat (B) at type locality of *Vietnamophryne occidentalis* sp. nov. in Doi Tung Mt., Chiang Rai Province, Thailand (Photos by P. Pawangkhanant and M. Naidaungchan)

Figure 5C, Figure 10, Figure 11; Table 3.

A: Dorsal view; B: Ventral view; C: Palmar view of right hand; D: Thenar view of left foot. Photos by P. Pawangkhanant.

**Holotype:** ZMMU A-5822, adult male in poor state of preservation, from a primary montane subtropical forest on limestone outcrops of Doi Tung Mt., Pong Ngam District, Chaing Rai Province, northern Thailand (N20.344°, E99.830°; elevation 1 050 m a.s.l.); collected on 5 April 2017 by Parinya Pawangkhanant at 1400 h from soil and leaf litter on the watershed of a steep mountain slope (Figure 11A) near a forest trail far from streams or rivers (Figure 11B).

**Diagnosis:** Assigned to the genus *Vietnamophryne* **Gen. nov.** based on morphological character traits and phylogenetic position in mtDNA genealogy (see Diagnosis of the new genus and Results). *Vietnamophryne occidentalis* **sp. nov.** can be distinguished from other congeners by the following combination of morphological features: (1) body size small, SVL of single male 20.5 mm; (2) body habitus stout, FLL/SVL and HLL/SVL ratios 62.7% and 140.3%, respectively; (3) head as long as wide, HW/HL ratio 99.0%; (4) snout short, obtuse in dorsal view, rounded in lateral view, shorter than eye length (85.5% of eye length); (5) eye medium-sized, eye length/snout-vent length ratio 12%; eye to nostril distance 6.7% of SVL; (6) tympanum comparatively small, rounded, 5.0% of SVL; located very close to eye (TED/SVL ratio 1.8%); (7) tips of digits rounded, not expanded in F1–F4, T1, T2, and T5, weakly expanded in T3 and T4; (8) first finger (F1) well developed, half of F2 length (1FL/2FL ratio 43.0%), relative finger lengths: I<II<IV<III, relative toe lengths: I<II<V<III<IV; (9) subarticular tubercles under fingers and toes weak, indistinct; (10) outer metatarsal tubercle absent, inner metatarsal tubercle small, rounded (4.2% of SVL); (11) skin of ventral surface completely smooth, skin of dorsal and lateral surfaces smooth, posteriorly with loosely scattered small flat tubercles present on dorsal surfaces of posterior dorsum and hindlimbs; (12) dorsomedial vertebral skin ridge distinct, well discernable on midline of dorsum and head; (13) dorsally dark brick-brown, lateral sides of head dark brown to black; ventrally orange-red with few dark brown flecks.

**Description of holotype:** Measurements of holotype are given in Table 3. Holotype in life is shown in Figure 5C and Figure 10. Body size small, SVL 20.5, in poor state of preservation (specimen was partially decayed prior to preservation, soft tissues absent from distal part of left hindlimb and middle part of belly); ventral surface of left thigh dissected 2.0 mm and partial femoral muscles removed. Body habitus stout (Figure 5C), head width equal to head length (HL/HW 99.0%); snout very short, truncate in dorsal view, rounded in lateral view (Figure 10A), snout length much shorter than eye length (SL/EL ratio 85.5%); eyes medium-sized (EL/SVL ratio 12.1%); eye to nostril distance 6.7% of SVL; eyes slightly protuberant in dorsal and lateral views (Figure 5C; Figure 10A, B), pupil round, horizontal; dorsal surface of head rather flat, canthus rostralis distinct, rounded; loreal region vertical; nostril rounded, lateral, located closer to tip of snout than to eye; tympanum well discernable, circular, comparatively small (TL/SVL ratio 5.0%), located very close to eye (TED/SVL ratio 1.8%); tympanic rim not elevated above skin of temporal area, supratympanic fold present, distinct and thick, rounded, glandular; vomerine teeth and spikes absent, single transverse palatal fold with smooth edge present across palate anteriorly to pharynx, tongue spatulate and free behind, lacking papillae, vocal sac opening absent.

Forelimbs comparatively long, almost half hindlimb length (FLL/HLL 44.7%); hand much shorter than lower arm, less than half forelimb length (HAL/FLL 43.9%); fingers comparatively long, slender, round in cross-section, first finger well developed, length slightly less than half of second finger (1FL/2FL 42.7%); relative finger lengths: I<II<IV<III (Figure 10C). Finger webbing and dermal fringes on fingers absent. First finger tip rounded, first finger well developed. Tips of three outer fingers II–IV rounded, not dilated, finger disks absent, terminal grooves absent; longitudinal furrow on dorsal surface of fingers absent; subarticular tubercles under fingers indistinct; nuptial pad absent; two palmar tubercles: inner palmar tubercle small, rounded; outer palmar tubercle rounded, slightly shorter than inner palmar tubercle (IPTL/OPTL 109.7%); palmar surface smooth, supernumerary palmar tubercles absent.

Hindlimbs short and thick, tibia length almost half of snout-vent length (TL/SVL 49.0%); tibiotarsal articulation of adpressed limb reaching eye level; foot length notably shorter than tibia length (FL/TL 82.0%); relative toe lengths: I<II<V<III<IV; tarsus smooth, tarsal fold absent; tips of toes rounded, tip of toe III slightly dilated, tip of toe IV notably dilated (Figure 10D), terminal grooves or dermal fringes on toes absent; toes rounded in cross-section; toe webbing absent between all toes; subarticular tubercles under toes indistinct; single metatarsal tubercle: inner metatarsal tubercle rounded, flattened (IMTL/SVL ratio 4.2%).

Skin on dorsal and dorsolateral surfaces smooth; rare small flat tubercles present on dorsal surfaces of hindlimbs and posterior dorsum (Figure 5C); dorsal surface of forelimbs smooth; upper eyelids smooth, supratympanic folds with low thick glandular ridges; ventral sides of trunk, head, and limbs completely smooth (Figure 10B); well-developed distinct dermal ridge present on midline of dorsal surface, running from tip of snout to cloacal area (Figure 5C; Figure 10A).

**Coloration of holotype in life:** Dorsally uniform dark brick-brown, continued on dorsal surfaces of limbs; rare small flat tubercles somewhat darker (dark brown) (Figure 10A); loosely scattered pustules on dorsal surfaces of posterior parts of dorsum and hindlimbs gray; dorsal surfaces of fore- and hindlimbs dark brick-brown; lateral sides of head dark brown (almost black); whitish mottling head sides or jaws absent (Figure 10A); canthus rostralis and supratympanic fold ventrally dark brown, dorsally brick-brown; ventrally bright orange-red with weak and rare dark brown marbling, denser on throat and ventral surfaces of hindlimbs (Figure 10B); fingers and toes dorsally dark brown, ventrally gray-brown to gray with occasional reddish blotches (Figure 10C, D). Pupil round, black, iris uniform dark brown (Figure 5C; Figure 10A).

**Coloration of holotype in preservative:** Coloration pattern unchanged after one year in ethanol; however, dorsal coloration changed to dark brown, reddish tint from dorsum and ventral surfaces faded completely; latter look yellowish-gray.

**Natural history notes:** The first record of *Vietnamophryne occidentalis* **sp. nov.** from Doi Tung Mt. was made by Akrachai Aksornneam on 10 February 2017. The specimen was encountered under a tree log at an elevation of ca. 1 000 m a.s.l. but was not collected. The holotype male specimen of the new species was encountered on 5 April 2017 during the day (1400 h) after heavy rain. The specimen was found at an elevation of ca. 1 050 m a.s.l. in leaf litter near a forest trail (Figure 11B) on the slope of Doi Tung Mt. with limestone outcrops (Figure 11A).

The climate of Doi Tung Mountain, Chiang Rai Province, is monsoonal with three distinct seasons: cool-dry from November to February, hot-dry from March to May, and rainy from May–June to November. The average annual rainfall is 2 500 mm at 1 200 m. Temperatures are lowest from November to February, with an average minimum at 500 m of 13 °C in January–February and 21 °C from June–August (Maxwell, 2007). At elevations above 1 000 m a.s.l., the typical montane forest canopy trees include: *Schima wallichii* (Theaceae), *Sarcosperma arboretum* (Sapotaceae), *Cinnamomum iners* (Lauraceae), *Balakata baccata* (Euphorbiaceae), and several Fagaceae (*Castanopsis armata*, *C. tribuloides*, and *Lithocarpus elegans*) (Maxwell, 2007).

As in other species of *Vietnamophryne* **Gen. nov.**, the biology of *Vietnamophryne occidentalis* **sp. nov.** remains completely unknown. Both known specimens were encountered during the day in soil under a large log or in leaf litter after heavy rain. As in other species of *Vietnamophryne* **Gen. nov.**, we assume that *Vietnamophryne occidentalis* **sp. nov.** has a secretive lifestyle and spends considerable time underground or in leaf litter. Despite intensive search efforts, only two specimens were encountered during two surveys. No calling activity was recorded during either survey, and reproductive biology and diet of *Vietnamophryne orlovi* **sp. nov.** remain unknown.

The associated species of amphibians and reptiles recorded in the area include: *Microhyla berdmorei* (Blyth, 1856), *Microhyla heymonsi* Vogt, 1911, *Sylvirana nigrovittata* (Blyth, 1856), *Rhacophorus rhodopus* Liu & Hu, 1960, *Theloderma albopunctatum* (Liu & Hu, 1962), *Theloderma gordoni* Taylor, 1962, *Acanthosaura lepidogaster* (Cuvier, 1829), *Pseudocalotes microlepis* (Boulenger, 1888), *Tropidophorus thai* Smith, 1919, *Oreocryptophis porphyraceus* cf. *porphyraceus* (Cantor, 1839), and *Ovophis monticola* (Günther, 1864).

**Comparisons:** For discrimination from other microhylid frogs occurring in Indochina, see “Comparisons with other Microhylidae genera inhabiting mainland Southeast Asia” above. For comparisons with *Vietnamophryne inexpectata* **sp. nov.** and *Vietnamophryne orlovi* **sp. nov.** see the “Comparisons” sections above.

**Distribution and biogeography:** To date, *Vietnamophryne occidentalis* **sp. nov.** is known only from its type locality in montane subtropical forest on limestone outcrops of Doi Tung Mt., Pong Ngam District, Chaing Rai Province, northern Thailand, at an elevation of ca. 1 050 m a.s.l.. Mt. Doi Tung belongs to a small mountain ridge located on the border between Chiang Rai Province of Thailand and Shan State of Myanmar; thus, the occurrence of the new species in adjacent parts of Myanmar is highly anticipated.

**Conservation status:** To date, *Vietnamophryne occidentalis* **sp. nov.** is known from a single locality based on one unvouchered record and the holotype specimen. Similar to other members of the genus *Vietnamophryne* **Gen. nov.**, it is likely that the new species has a secretive semi-fossorial biology. Additional focused survey efforts in adjacent parts of Thailand and Myanmar are required to clarify the range and population status of *Vietnamophryne occidentalis* **sp. nov.** Given the available information, we suggest *Vietnamophryne occidentalis* **sp. nov.sp. nov.** be considered as a Data Deficient (DD) species following IUCN’s Red List categories (IUCN Standards and Petitions Subcommittee, 2016).

**Etymology:** The specific name “*occidentalis*” is a Latin adjective in the nominative singular meaning “western”; referring to the type locality of the new species in western Indochina (Chiang Rai Province of Thailand) – to date, the westernmost area where members of the subfamily Asterophryinae are recorded.

**Suggested common names:** We recommend the following common names for the new species: “Chiang Rai Dwarf Frog” (English) and “Eung Tham Khaera Chiang Rai” (Thai).

## DISCUSSION

In this work, we report on the discovery of a new lineage of Asterophryinae microhylid frogs from Indochina. *Vietnamophryne* is a genus of small miniaturized frogs. Although the specimens were mostly recorded in soil or under large tree-trunks, suggesting a semi-fossorial lifestyle, they lack obvious adaptations for digging. Due to their secretive underground biology, they have been encountered by herpetologists only rarely and have remained almost unnoticed despite 200 years of herpetological studies in Indochina. Even with our intensive effort, we were unable to collect additional specimens of the three new species from the three localities in Vietnam and Thailand. It is anticipated, however, that members of the genus *Vietnamophryne* will be discovered in other parts of Indochina, including central and northern Vietnam, Laos, and northern Myanmar. Our work calls for intensification of focused herpetological surveys combined with molecular analyses to further our understanding of amphibian biodiversity in Indochina. Intensive examination of museum herpetological collections also might result in the discovery of Asterophryinae specimens, as these frogs may have been misidentified as juveniles of other microhylid species in previous work.

As predicted by Kurabayashi et al. (2011), *Vietnamophryne* represents an ancient lineage of Asterophryinae differentiation distributed deep in mainland Southeast Asia (northern Indochina). Here, *Vietnamophryne* was reconstructed as a sister lineage to *Siamophryne* from southern Indochina (north of Isthmus of Kra, Figure 1; Suwannapoom et al., 2018), and the clade joining the two latter genera was determined to be a sister clade to *Gastrophrynoides* from Sundaland (south of Isthmus of Kra, Figure 1). Thus, our discovery of the genus *Vietnamophryne* and three constituent species brings the number of Asterophryinae species reported for Indochina to five, and illustrates that the basal cladogenetic events within the subfamily most likely occurred on the Eurasian landmass, followed by subsequent radiation. This further supports the “out of Indo-Eurasia” scenario of Kurabayashi et al. (2011): according to their divergence estimates, the common ancestor of Asterophryinae diverged from other Microhylidae lineages during the late Cretaceous (possibly on the Indian subcontinent), and the basal split within the subfamily occurred during the Eocene (∼48 Ma, Kurabayashi et al., 2011). Our data suggest that this split, separating the ancestor of *Gastrophrynoides*+*Siamophryne*+*Vietnamophryne* from the ancestor of all other “core” Australasian Asterophryinae, most likely took place in Indochina. While the “core” Asterophryinae ancestors dispersed further eastwards, crossed the Wallace line, colonized the Australasian landmass, and diversified during the late Oligocene (∼25 Ma, Rivera et al., 2017), the cladogenesis within the Eurasian Asterophryinae was less intensive. Divergence within the genus *Vietnamophryne* was, most likely, a comparatively recent event due to the small genetic distances observed among species.

A similar biogeographic “out of Indochina to Australasia” pattern has been reported in several other taxonomically diverse groups of amphibians and reptiles. For example, Yan et al. (2016) demonstrated that the speciose frog family Ceratobatrachidae (Natatanura) originated in the eastern Himalayas and Tibet, from where it colonized and subsequently radiated to the islands of the Australasian archipelago. Wood et al. (2012) reported a generally similar biogeographic pattern for the most diverse genus of geckoes (*Cyrtodactylus*), suggesting that the genus formed in the eastern Tibet-Himalayan region, from where it colonized the tropical areas of South and Southeast Asia. According to this scenario, Indochina served as a local diversification center of *Cyrtodactylus*, with several waves of dispersal allowing this genus to colonize Sundaland, Lesser Sunda Islands, the Philippines, Papua New Guinea, and adjacent Australasian islands and northern Australia (Wood et al., 2012). Hence, the biogeographic scenarios for at least two of most speciose Australasian frog families and the most speciose gecko genus argue an initial origination and cladogenesis in mainland Southeast Asia followed by dispersal into the Australasian archipelago and subsequent radiation. Our study further suggests that the Indochinese Peninsula played a key role in the formation of the herpetofauna of Southeast Asia and Australasia.

Our dataset on the “core” Asterophryinae was based on sequences obtained from earlier studies (see Table 1 for details), and our results on phylogenetic relationships among members of the Asterophryinae 1 clade were generally in accordance with previously published data. This speciose group underwent adaptive radiation in the Australo-Papuan region, with members of the Asterophryinae 1 clade demonstrating various lifestyles, including arboreal, scansorial, terrestrial, burrowing (fossorial), and semi-aquatic (Rivera et al., 2017). This adaptive radiation has led to numerous homoplasies and reversal shifts in the evolution of morphological characteristics, thus hampering the progress of generic taxonomy based solely on morphological evidence (Burton, 1986; Köhler & Günther, 2008; Menzies, 2006; Rivera et al., 2017; Zweifel, 1972). The multilocus analysis of phylogenetic relationships following wide sampling of New Guinean asterophryines by Rivera et al. (2017) showed that basal radiation of Asterophryinae occurred in a narrow timeframe between 20–27 Ma and was accompanied by numerous ecomorphological shifts. Rivera et al. (2017) pointed out 11 asterophryine genera as paraphyletic, suggesting that in most cases they can be brought into monophyly by collapsing genera (*Albericus* is synonymized with *Choerophryne*; *Oreophryne* clade 3 is synonymized with *Aphantophryne*; *Genyophryne*, *Oxydactyla* and *Liophryne* are synonymized with *Sphenophryne*). However, some of the groups in Rivera et al. (2017) tree got low or no node support, thus hampering further taxonomic decisions (e.g., *Austrochaperina* and *Copiula*). Though based on limited taxon and molecular sampling, our analysis indicated that *Sphenophryne thomsoni*, previously assigned to the genus *Genyophryne*, was a sister lineage to the clade that united *Cophixalus* and *Choerophryne*, thus suggesting that the synonymization of *Genyophryne* with *Sphenophryne* may be premature. It is obvious that generic taxonomy of Asterophryinae is still in a state of flux and further molecular and morphological research is needed to achieve a better taxonomic hypothesis for this group.

Due to the paucity of observations and very limited sampling, the natural history of *Vietnamophryne* remains almost completely unknown. Our data suggest that the new genus prefers undisturbed evergreen forests with a secretive, possibly semi-fossorial, lifestyle, and spends substantial time sheltering in leaf litter and soil. We have no information on diet, enemies, reproduction, or life cycle of the new genus. All members of the “core” Asterophryinae clade inhabiting Australasia, for which breeding has been observed, are known to have direct development – i.e., a life cycle with metamorphosis taking place within the egg (Günther et al., 2012b; Menzies, 2006). However, the recently discovered *Siamophryne* has a peculiar tadpole, which is, to date, the only record of the existence of a larval stage for Asterophryinae (Suwannapoom et al., 2018). The reproductive biology and development of *Gastrophrynoides* also remain unknown (Chan et al., 2009; Parker, 1934). Superficially, the miniaturized *Vietnamophryne* resembles some small ground-dwelling genera of the “core” Asterophryinae, which exhibit direct development. However, as all collected specimens of *Vietnamophryne* were males, we cannot speculate on the possible reproduction mode of the new species. Due to the ancient divergence and phylogenetic history, morphological differences, and peculiarities of life cycle (e.g., larval development in *Siamophryne*), we cannot exclude that the taxonomic status of the Eurasian Asterophryinae lineage might be reconsidered in the future.

Our work adds a new genus and three new species of frogs to the batrachofauna of Indochina. The real extent of distribution of the species described herein is unknown and requires further study. Undisturbed montane forests of eastern and northern Indochina cradle one of the richest herpetofaunas in the world (Poyarkov et al., *unpublished data*). However, deforestation is a growing threat in Indochina, especially in Vietnam (Meyfroidt & Lambin, 2008), and habitat loss and modification are widely recognized as major threats to amphibians in Southeast Asia. Forest specialist species restricted to primary undisturbed broadleaf evergreen montane forests would be especially vulnerable to changes in their environment. Further field survey efforts and molecular taxonomic studies are essential for the effective estimation and conservation management of amphibian biodiversity in Indochina.
